# Permeability Data of Organosulfur Garlic Compounds Estimated by Immobilized Artificial Membrane Chromatography: Correlation Across Several Biological Barriers

**DOI:** 10.3389/fchem.2021.690707

**Published:** 2021-09-20

**Authors:** Daniela Andrea Ramirez, María Fernanda Federici, Jorgelina Cecilia Altamirano, Alejandra Beatriz Camargo, Juan María Luco

**Affiliations:** ^1^ Instituto de Biología Agrícola de Mendoza (IBAM), CONICET-Mendoza, Mendoza, Argentina; ^2^ Laboratorio de Cromatografía para Agroalimentos, Facultad de Ciencias Agrarias, Universidad Nacional de Cuyo, Mendoza, Argentina; ^3^ Facultad de Ciencias Exactas y Naturales, Universidad Nacional de Cuyo, Mendoza, Argentina; ^4^ Instituto Argentino de Nivología, Glaciología y Ciencias Ambientales (IANIGLA), CONICET-Mendoza, Mendoza, Argentina; ^5^ Área de Química Analítica, Facultad de Química, Bioquímica y Farmacia, Universidad Nacional de San Luis, San Luis, Argentina

**Keywords:** QSAR, IAM, permeability, biological barrier, organosulfur compounds

## Abstract

Among healthy vegetables, those of the genus *Allium* stand out. Antioxidant and anti-inflammatory properties have been associated with these vegetables, attributed mainly to organosulfur compounds (OSCs). In turn, they are linked to a protective effect counteracting cardiovascular disease development. Now, to really ensure the bioactive efficacy of the said compounds once consumed, it is necessary to previously evaluate the ADME (absorption, distribution, metabolism, and excretion) profile. Alternatively, *in vitro* and *in silico* methods attempt to avoid or reduce experimental animals’ use and provide preliminary information on drugs’ ability to overcome the various biological barriers inherent in the ADME process. In this sense, *in silico* methods serve to provide primary information on drugs’ bioavailability mechanisms. High-performance liquid chromatography (HPLC) using a stationary phase composed of phospholipids, the so-called immobilized artificial membrane (IAM), has been widely recognized as a valuable alternative method to extract and quantify information about the structure and physicochemical properties of organic compounds which are extensively used in studies of quantitative structure–activity relationships (QSARs). In the present study, the chromatographic capacity factors (log *k’* (IAM)) for 28 OSCs were determined by IAM-HPLC. In order to evaluate the ability of the IAM phase in assessing lipophilicity of the compounds under study, several quantitative structure–retention relationships (QSRRs) were derived from exploring fundamental intermolecular interactions that govern the retention of compounds under study on IAM phases. As expected, the hydrophobic factors are of prime importance for the IAM retention of these compounds. However, the molecular flexibility and specific polar interactions expressed by several electronic descriptors (relative negative charge, RNCG, and Mulliken electronegativity) are also involved. We also evaluated the IAM phase ability to assess several ADME parameters for the OSCs under study obtained using the SwissADME web tool integrated into the SwissDrugDesign workspace and the PreADMET web tool. The human gastrointestinal absorption (HIA), blood–brain barrier (BBB) permeation, and skin permeability were investigated through QSAR modeling, using several chemometric approaches. The ADME properties under study are strongly dependent on hydrophobic factors as expressed by log *k’*(IAM), which provide evidence for the great potential of the IAM phases in the development of QSAR models.

## Introduction

The current guidelines recommend diets rich in fruits and vegetables as an important factor in reducing the risk of developing chronic diseases (Yu et al., 2018). Among healthy vegetables, those of the genus *Allium* stand out. Particularly, abundant literature supports garlic and its by-products' health-promoting effects associated with bioactive compounds present in their matrix (Corzo-Martinez et al., 2007; Santhosha et al., 2013; Ramirez et al., 2021). Amid these bioactivities, cardiovascular protection is one of the most well-known beneficial effects related to garlic or garlic-based products' intake (Banerjee and Maulik, 2002; Santhosha et al., 2013; Schwingshackl et al., 2016). In this sense, evidence has shown that organosulfur compounds (OSCs) present in different garlic matrices interfere against inflammation, oxidative stress, obesogenic effects, and mitochondrial dysfunction (Quesada et al., 2020) by multiple actions, such as modifying signaling pathways that trigger chronic diseases.

Now, if we focus on the bioactive efficacy, we must first ensure that the phytochemical compounds manage to reach the target sites within the body where they will exert their biological effect, so it is necessary to previously evaluate *in vivo* the ADME (absorption, distribution, metabolism, and excretion) profile of the said compounds (Rein et al., 2013). Considering the previously mentioned biological steps, cellular permeability of bioactive compounds is one of the key physicochemical parameters to be considered when selecting potential therapeutic candidates (Gozalbes et al., 2011). To estimate cellular permeability there have been several absorption models that are proposed, including *cell-based* models, using the human colorectal adenocarcinoma (Caco-2) cell line and the Madin-Darby Canine Kidney (MDCK) cell line and *tissue-based* models, such as *in situ* rat intestinal perfusion or the Ussing chamber system employing rat or human intestinal tissues (Berben et al., 2018). Although these models are useful in terms of permeability prediction, they have several drawbacks, including high-cost and complex experimental procedures as well as ethical issues associated with animal-based assays (Grumetto et al., 2015a).

A recent work from our research group has determined that intestinal absorption proved to be a limiting step for OSC availability (Torres-Palazzolo et al., 2018). However, it is necessary to delve deeper into the structural basis of the permeability stage mechanisms based on a molecular level (Butina et al., 2002). Alternatively, computational approaches (*in silico*) serve to provide primary information on drugs’ bioavailability mechanisms (Yen et al., 2005). In this context, alternative methods, such as chromatographic ones, employing immobilized artificial membrane (IAM) chromatographic columns, have been widely recognized as a valuable tool to model processes in the biophase since the components of both chromatographic and biological systems are comparable. This is supported by the fact that some biological processes, such as passive cellular diffusion, have much in common with the processes that take place in some chromatographic separations (Escuder-Gilabert et al., 2003). To delve deeper into the physicochemical processes that are involved in cellular permeability, the information resulting from chromatographic data using membrane-like systems, such as stationary phases containing phosphatidylcholine or similar lipid-like ligands, is used to develop statistical models called quantitative structure–activity relationship (QSAR) models, using several chemometric tools.

Based on the above-mentioned models, the chromatographic capacity factors (log *k*’ (IAM)) for 28 OSCs were determined by IAM-HPLC and various quantitative structure–retention relationships (QSRRs) were derived to explore the fundamental intermolecular interactions that govern the retention of compounds under study on the IAM phases. Thus, the partial least squares (PLS) approach was used to model the IAM chromatographic data determined for these compounds. The molecular characterization was carried out by calculating numerous nonempirical descriptors, which were subsequently used to construct QSRR models.

On the other hand, we also attempted to evaluate the IAM phase ability to assess several ADME parameters for the OSCs under study using the SwissADME web tool integrated into the SwissDrugDesign workspace and the PreADMET web tool. The human gastrointestinal absorption (HIA), blood–brain barrier (BBB) permeation, and skin permeability were investigated through QSAR modeling, using several chemometric approaches.

## Materials and Methods

### Organosulfur Compounds Under Study, Reactives, and Analytical Grade Solvents

Garlic cysteine compounds, like S-methyl-L-cysteine and S-propyl-L-cysteine, were purchased from Sigma (St. Luis, MO, United States), whereas S-allyl-L-cysteine (SAC) was purchased from LKT Laboratories Inc. (St. Paul, MN, United States). S-Alk(en)yl-L-cysteine sulfoxides (ACSOs) such as (+)-S-methyl-L-cysteine sulfoxide (methiin), (+)-S-trans-1-propenyl-L-cysteine sulfoxide (isoalliin), and diallyl thiosulfinate (allicin) were synthesized in our laboratory as previously described (Thomas and Parkin, 1994). Alliin, (+)-S-allylcysteine sulfoxide, was purchased from Extrasynthese (Lyon-Nord, France).

Sulfide-based compounds, such as diallyl sulfide (DAS), diallyl disulfide (DADS), and diallyl trisulfide (DATS), were purchased from Sigma-Aldrich (St. Luis, MO, United States). Ethyl disulfide (ES), furfuryl disulfide (FuDS), propyl disulfide (PDS), allyl methyl sulfide (AMS), and dimethyl disulfide (DMDS) were obtained from Aldrich (United States). Ethyl sulfide (ES) was obtained from Fluka^®^ (Munich, Germany). Allyl mercaptan was purchased from Sigma-Aldrich (St. Luis, MO, United States).

E-Z Ajoene and vinyldithiins compounds were synthesized following a previously reported procedure by our research group (Ramirez et al., 2017). Then, the synthesized OSCs were concentrated under reduced pressure and characterized by UV-spectroscopy and GC–MS analysis.

Allyl ITC (AITC), sulforaphane (SF), indole-3-carbinol (I3C), and phenyl ITC (PITC) were obtained from Sigma-Aldrich (St. Luis, MO, United States). Phenethyl ITC (FITC), S-sulforaphene (SE), and benzyl ITC (BITC) were purchased from LKT Laboratories Inc. (St. Paul, MN, United States).

Potassium phosphate monobasic and dibasic (anhydrous) were of PA grade and were purchased from Biopack.

Acetonitrile (ACN) was of chromatographic grade and was purchased from Merck (United States). Ultrapure water (18 MΩcm) was obtained from a Milli-Q water purification system (Millipore, France).

### Chromatographic Conditions and Instrumentation

Chromatographic analysis was performed using a Shimadzu LC 20A chromatograph coupled to a diode array detector, operated at a range of 210–260 nm, depending on the analyzed compound. A biomimetic commercially available column was used in all experiments: IAM. PC.MG12 µm (150 × 4.6 mm) was purchased from Regis Chemical Company (Morton Grove, IL).

OSC separation conditions under IAM-HPLC were adapted from Luco et al. (2003) and Barbato et al. (1998), with modifications. The chromatographic mode consisted in an isocratic elution used as the mobile phase ACN/0.035 M phosphate buffer −pH 6.8− (30:70 (v/v)) at 0.5 ml min^−1^. The column dead time (T_0_) was estimated by ascorbic acid retention time which was measured at 210 nm. The obtained retention data were used to calculate the chromatographic parameter corresponding to the capacity factor *k’* as follows: *k’* = (T_r_–T_0_)/T_0_. All capacity factors given represent the mean of 2–4 determinations of each sample solution.

For this study, we considered 28 phytochemical compounds containing sulfur-based groups. Supplementary Table S1 shows their name and chemical structure. Most of these compounds are garlic-derived substances with bioactive functions. However, we also included seven phytochemicals—sulforaphane, sulforaphene, I3C, allyl ITC, phenethyl ITC, phenyl ITC, and benzyl ITC—that correspond to glucosinolates degradation products, such as isothiocyanates and indole-3-carbinol. We chose them because of their bioactive functionality and their similar metabolism within the plants and human body. Each analytical standard stock solution was prepared in the mobile phase at concentrations ranging from 100 to 800 µg ml^−1^, depending on the solubility of the compound and their DAD detector response. Peak identification in samples was carried out by comparing retention times with reference standards.

### Molecular Descriptors and ADME Properties

To characterize each OSC, a set of molecular descriptors were calculated using different software packages, including Dragon vs. 3.0 and the Online Chemical Modeling Environment (OCHEM) web platform. As for indicators of the molecular size and structural descriptors, the following were considered: molar volume (Vm), molecular weight (MW), rotatable bonds, H-donors, H-acceptors, and TPSA (topological polar surface area). To explain lipophilicity effects, several calculated partition coefficients were obtained: log D, iLOGP, XLOGP3, WLOGP, MLOGP, Silicos-IT Log P, and Consensus Log P. These parameters were obtained from different online web tools, such as SwissADME (developed and maintained by the Molecular Modeling Group of the Swiss Institute of Bioinformatics) and Chemicalize^©^ 1998-2021 (ChemAxon, Ltd.).

Another group of structural descriptors included quantum chemical indexes obtained by the HyperChem package (release 7.5 for Windows). Three-dimensional molecular structures were built using two different software: the MM + molecular mechanics potential energy function employing the HyperChem package and on the other side the CORINA Classic online service. In a follow-up procedure, complete optimization of the geometrical parameters was carried out by using the PM3 method, implemented in the standard version of MOPAC 6.0. The following indices obtained from molecular orbital calculations were considered: total energy (E_total_), heat of formation (ΔHf), energy of highest occupied molecular orbital (HOMO), energy of lowest unoccupied molecular orbital (LUMO), dipole moment (µ), absolute total charge (Qtotal), the most positive and the most negative absolute charges (q_p,max_, q_n,max_), and the positive and negative relative charge (RNCG and RPCG). Furthermore, other descriptors obtained from Dragon software were considered such as Mulliken electronegativity, LDip, and several WHIM descriptors. Multivariate analyses including principal components analysis (PCA) and a stepwise multiple regression procedure, based on the algorithms forward selection and backward elimination, were used for the inclusion or rejection of descriptors in the screened models.

Finally, to assess permeability parameters for the OSCs under study, several calculated properties were obtained from the SwissADME web tool and the PreADMET web-based application (^©^ 2005-2017 BMDRC. | Designed by Y.-M. Kang). The human gastrointestinal absorption (HIA), blood–brain barrier (BBB) permeation, and skin permeability (Log *K*_P_) were investigated.

To ensure the reliability of the *in silico* data about HIA, BBB passage, and *Kp*_skin_ calculated from the SwissADME and PreADMET web platforms, the predictor space of each platform was analyzed. By doing so, the applicability domain (AD) of each QSAR model which serves to calculate the prediction values offered in the web tools can be tested. *In silico* prediction systems feature the capability to evaluate if a query molecule is a part of the developed model’s AD or not and, therefore, gave an improved sense of reliability at the individual prediction level (Kar et al., 2018). For this, a series of physicochemical descriptors were calculated, both for each of the compounds that make up the predictor space associated with each permeation response (241 compounds from the data set to build the HIA permeation model (Zhao et al., 2001), 260 compounds from the data set were used to calculate BBB passage (Daina and Zoete, 2016), and 96 compounds from the data set were used to calculate *Kp*_skin_, as well as for our 28 OSCs. From these data, we then performed a PCA, and it was evaluated whether our OSCs belong to the predictive space generated by the compounds used in such platforms.

### Statistical Methods

A multivariate analysis was used primarily. Multiple regression analysis (MLR) and partial least squares projections (PLS) were the selected methods to build different statistical models between IAM chromatographic parameters and the structural descriptors. The determination of the significant number of PLS components was made by cross-validation. MLR analysis was performed using MINITAB^®^ Release 17, whereas PLS analysis was carried out using SIMCA-P 7.01 software obtained from Umetri AB, Umea, Sweden. To avoid overestimations or difficulties in the interpretation of the resulting models, pairs of variables with r ≥ 0.75 were classified as intercorrelating ones, and only one of these was included in the screened model. The predictive ability of the models were evaluated by the squared correlation coefficient (*R* ^2^) and the Fisher *F* statistic (*F*). In PLS models, we also evaluated the cross-validation coefficient (Q^2^), which is based on the prediction error sum of squares (PRESS). Considering the QSAR models to assess permeability parameters, we used TableCurve 2D^®^ to build a nonlinear curve-fitting model for HIA vs. log *k’*IAM, MINITAB^®^ Release 17 to carry out a discriminant analysis to assess BBB permeation, while skin permeation was evaluated using a PLS analysis (SIMCA-P 7.01 software). Principal component analysis to check OSCs belonging to the predictor space of each permeation response, according to the different web platforms, was carried out using MINITAB ^®^ v.17.

## Results

### OSC Lipophilicity Determination Using IAM and Physicochemical Parameters

First, we evaluated the IAM phase ability in assessing OSC lipophilicity. For this, we studied to what extent the log *k’*IAM values were related to the calculated values of the partition coefficient log P, obtained from the SwissADME web tool. Table 1 shows the chromatographic data and the corresponding physicochemical parameters of the compounds under study. In this case, we built a MLR model based on iLOGP (implicit Log P). This parameter is a calculated value corresponding to the n-octanol/water partition coefficient descriptor based on the GB/SA approach. It has been proven to be an efficient physicochemical parameter showing a strong linear correlation between the computed solvation free energy in implicit solvents and the experimental log P_o/w_ on more than 17,500 molecules (Daina et al., 2014). The regression equation using the whole set of compounds was as follows:

$$\begin{array}{l} \log k’\left(\mathrm{IAM}\right)=-2\mathrm{.08}\left(0.2484\right)+1\mathrm{.02}\left(0.1284\right)\mathrm{iLOGP}\\ \left(0\mathrm{.000}\right)\;\left(0.000\right)\\ R^{2}=0.721;R^{2}\left(cv\right)=0.683;s=0.444;n=28;F=67.29;W_{iLOGP}=0.8492 \end{array}$$ (1)

In Eq. 1 and the following equations, *n* is the number of compounds, *s* is the standard deviation, *R* ^2^ is the squared correlation coefficient, *R* ^2^_*(cv)*_ is the squared cross-validation coefficient, and *F* is the Fisher F statistic. Values in parentheses correspond to the standard deviations and *p-values* of coefficients, and the term *W* represents the standardized regression coefficient.

**TABLE 1:** IAM chromatographic data, ADME parameters, and molecular descriptors.

OSCs	Retention time (min)	Log (*k'*IAM)^a^	HIA%	Log *K*_*P*_ (cm/s)	BBB permeant	p*K*a1 (acidic)	p*K*a2 (basic)	Rotatable bonds	TPSA	iLOGP	RNCG_1	ElecMullk	Tm_1	TE1	TPSA_1
Propyl disulfide	33.44	0.913	98.141	−5.30	Yes	^b^	^b^	5	50.60	2.63	0.221	5.624	7.281	4.149	0.000
Isoalliin	4.31	−0.734	73.041	−9.83	No	1.84	8.44	4	99.60	0.88	0.293	4.735	8.189	11.123	177.913
S-allyl-l-cysteine	3.93	−1.102	81.972	−8.75	No	2.53	9.14	5	88.62	1.22	0.285	4.581	7.758	6.222	163.722
Alliin	3.71	−1.710	73.041	−9.89	No	1.84	8.45	5	99.60	0.55	0.283	5.071	8.386	11.192	172.185
S-propyl-l-cysteine	3.72	−1.645	78.546	-8.59	No	2.62	9.14	5	88.62	1.18	0.283	4.724	9.452	6.656	161.751
S-methyl-l-cysteine	3.81	−1.322	74.078	−9.06	No	2.44	9.15	3	88.62	0.74	0.322	4.745	5.43	5.675	161.734
Methiin	3.68	−1.922	62.663	−10.25	No	1.61	8.45	3	99.60	0.24	0.314	5.021	5.805	10.051	265.833
2-vinyldithiin	9.92	0.237	98.169	−5.55	Yes	^b^	^b^	1	50.60	1.98	0.244	4.695	4.263	3.595	0.000
Diallyl disulfide	16.68	0.554	98.169	−5.63	Yes	^b^	^b^	5	50.60	2.49	0.166	5.630	6.75	3.512	0.000
Ethyl disulfide	13.21	0.420	97.832	−5.88	Yes	^b^	^b^	3	50.60	2.17	0.276	5.615	4.424	2.883	0.000
Dimethyl disulfide	7.14	−0.017	97.185	−5.62	Yes	^b^	^b^	1	50.60	1.69	0.473	5.688	2.993	1.944	0.000
Ethyl sulfide	6.54	−0.099	100.000	−5.47	Yes	^b^	^b^	2	25.30	1.92	0.282	4.609	3.649	2.452	0.000
Allyl methyl sulfide	3.99	−1.021	100.000	−5.76	Yes	^b^	^b^	2	25.30	1.77	0.314	4.520	3.663	2.490	0.000
E-Ajoene	7.46	0.022	99.314	−6.52	No	14.9	^b^	8	86.88	2.74	0.285	5.637	16.744	10.291	41.722
Z-Ajoene	7.41	0.015	99.314	−6.52	No	14.9	^b^	8	86.88	2.74	0.287	5.681	13.945	10.421	36.282
Diallyl sulfide	9.71	0.222	100.000	−5.46	Yes	^b^	^b^	4	25.30	2.11	0.170	4.482	6.067	3.223	0.000
Methyl Propyl Trisulfide	27.12	0.810	98.302	−5.65	Yes	^b^	^b^	4	75.90	2.33	0.333	5.945	6.979	3.055	0.000
Furfuryl disulfide	22.44	0.713	98.910	−6.19	Yes	^b^	^b^	5	76.88	2.62	0.147	5.504	10.911	4.647	34.289
Allicin	5.06	−0.409	98.312	−6.36	Yes	^b^	^b^	5	61.58	1.95	0.339	5.686	6.92	6.924	36.453
Allyl mercaptan	5.95	−0.197	96.865	−5.93	Yes	10	^b^	1	38.80	1.51	0.333	4.643	3.137	1.532	0.000
Sulforaphane	4.75	−0.514	97.884	−6.38	No	^b^	0.87	5	80.73	2.11	0.384	4.963	12.924	8.191	67.965
Sulforaphene	4.74	−0.521	98.344	−6.32	No	^b^	0.66	4	80.73	2.17	0.379	5.051	12.5	7.732	62.692
Diallyl Trisulfide	25.6	0.781	98.996	−5.51	Yes	^b^	^b^	6	75.90	2.65	0.165	5.970	8.334	3.549	0.000
Indole-3-carbinol	5.91	−0.204	89.057	−6.45	Yes	15.1	^b^	1	36.02	1.36	0.246	4.234	5.623	5.450	78.717
Allyl isothiocyanate	8.42	0.118	98.086	−5.19	Yes	^b^	0.65	2	44.45	1.93	0.409	4.891	6.325	1.620	57.462
Phenethyl isothiocyanate	20.38	0.663	97.812	−4.83	Yes	^b^	1.36	3	44.45	2.47	0.167	4.904	11.256	3.006	19.910
Phenyl isothiocyanate	23.9	0.746	98.001	−4.80	Yes	^b^	−1.83	1	44.45	2.09	0.236	4.813	7.947	2.192	53.721
Benzyl isothiocyanate	17.18	0.571	97.906	−4.97	Yes	^b^	0.34	2	44.45	2.19	0.193	4.883	7.262	2.574	19.380

a For an explanation of the symbols, refer to the text.

b Nonionizable compound.

**FIGURE 1:**
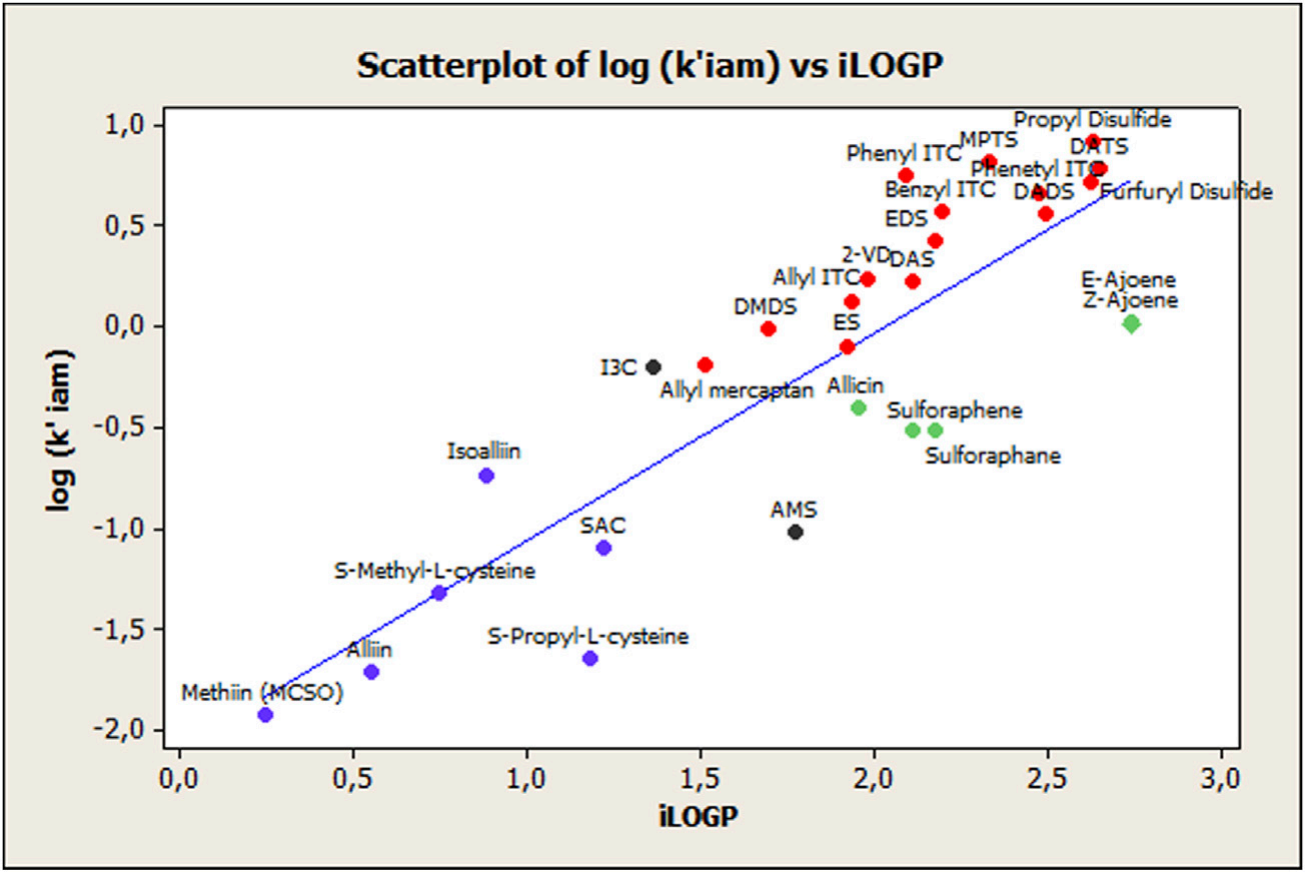
Relationship between log *k'* (IAM) and iLOGP for the 28 compounds shown in Table 1. Key:
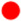
Nonionizable organosulfur compounds
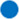
Ionizable organosulfur compounds
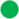
Compounds with sulfoxides
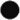
Unusual observations.

The model statistical quality is suitable as seen in the quality of fit and predictive ability, expressed by *R* ^2^ and *R*2_*cv*_ coefficients, respectively. However, some aspects should be considered to further improve the statistics of Equation 1. The relationship between the log *k’*(IAM) and iLOGP values is depicted in Figure 1. There, it is possible to distinguish different subgroups of compounds, marked by different colors, corresponding to nonionizable OSCs (mostly sulfide-based compounds), ionizable OSCs (ACSOs and cysteine-containing compounds), sulfoxide-based compounds (allicin, sulforaphane/ene, and E-Z-ajoene), and two special cases (I3C and AMS). This suggests a divergence between the different compounds that may be due to the sulfoxide groups’ different interactions, both with phosphatidyl choline in the IAM phase as well as with octanol, when we consider the octanol–water partition. These interactions will be explained in more detail in the next section, and they will be discussed accordingly.

### QSRR Models to Assess OSC Interactions With the IAM Phase

Several QSRR equations were built to evaluate the fundamental intermolecular interactions that govern the retention of the different OSCs on the IAM phases. As shown in the previous section, lipophilicity is an essential factor to be considered in the quantitative structure–retention relationship (QSRR) models. Nevertheless, since lipophilicity can be described by the contribution of two components, namely molecular size and molecular polarity, models that incorporated both molecular properties were examined. The best regression equation that included the iLOGP parameter was as follows:

$$\begin{array}{l} \log k’\left(\mathrm{IAM}\right)=-3\mathrm{.40}\left(0.4967\right)+0\mathrm{.633}\left(0.1188\right)\mathrm{Mulliken Electronegativity}\\ \left(0\mathrm{.000}\right)\;\left(0.000\right)\\ -2\mathrm{.61}\left(0\mathrm{.5974}\right)\mathrm{RNCG\_1}+0\mathrm{.876}\left(0\mathrm{.0807}\right)\mathrm{ILOGP-0}\mathrm{.201}\left(0\mathrm{.0251}\right)\mathrm{Rotatable bonds}\\ \left(0\mathrm{.000}\right)\;\left(0.000\right)\;\left(0\mathrm{.000}\right)\\ R^{2}=0.936;R^{2}\left(cv\right)=0.907;s=0.226;n=28;F=84.1;W_{iLOGP}=0.726;W_{Mullk.Elec.}=0.362;W_{Rot.Bonds}=-0.481;W_{RNCG\_1}=-0.252 \end{array}$$ (2)

This equation is highly significant, and there are no strong intercorrelations among the selected molecular descriptors, which is fundamental to reach a correct physicochemical interpretation. An interesting point to highlight in this equation is an improvement in the statistical parameters when compared to Eq. 1, which suggests not only a better predictive ability but also, as hypothesized, that specific interactions regarding OSC behavior in the IAM phase precisely emerge from the other terms that are added to the model, besides iLOGP.

As an alternative way to evaluate the reliability and robustness of the QSRR model, we carried out a different statistical approach using the same descriptors included in the MLR model. Accordingly, we performed the analysis of molecular descriptors using partial least squares regression. The statistical significance of the QSRR-PLS derivative models was evaluated by means of the variance of the matrices X and Y (R2X and R2Y), the square correlation coefficient (*R* ^2^), standard deviation (RMSS), and the statistical F. The predictive ability was evaluated by the cross-validation coefficient (Q) which is based on the prediction error sum of squares (PRESS). The PRESS statistic is computed as the squared differences between observed and predicted values when the observations are kept out of the derived model (Luco et al., 2003).

**FIGURE 2:**
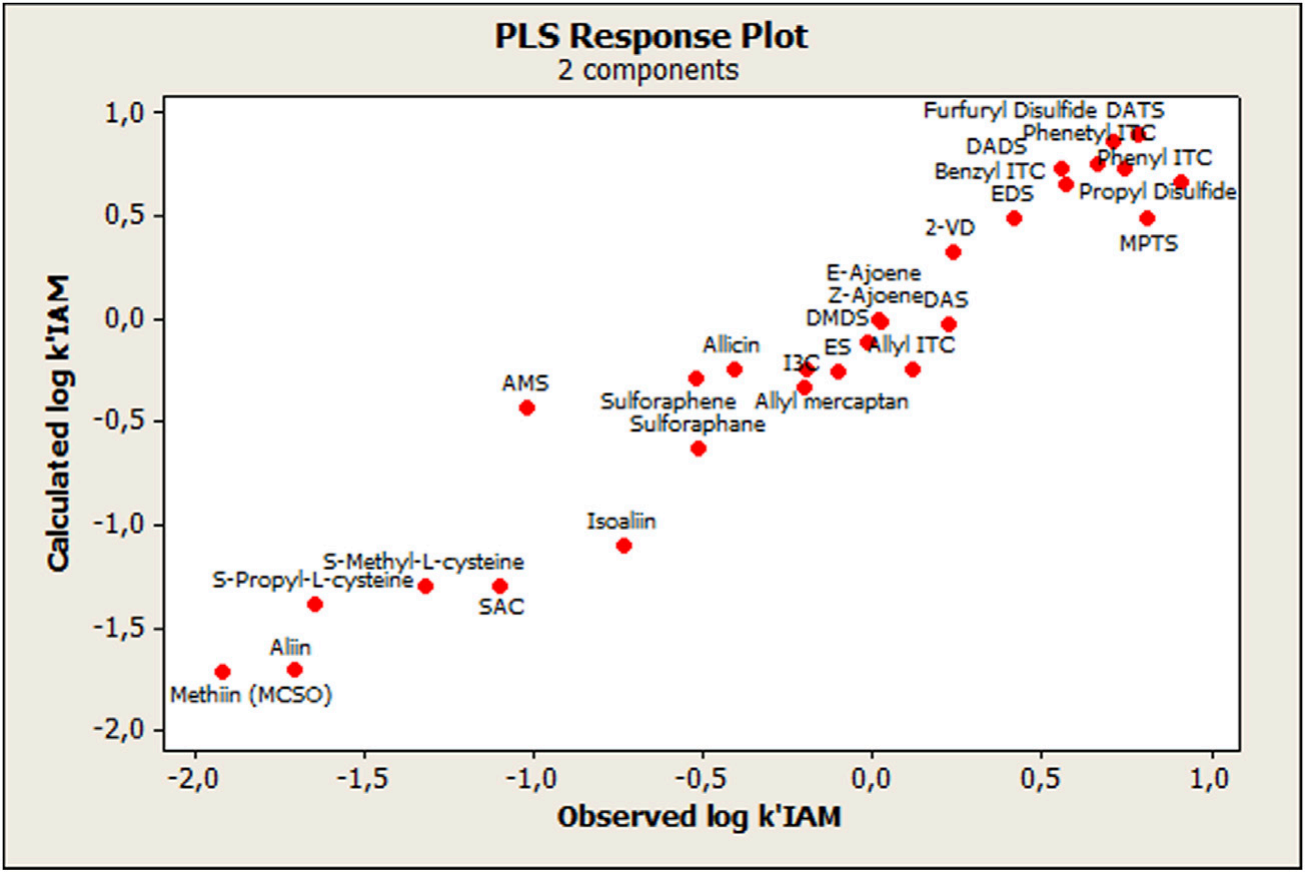
Relationship between observed and calculated values using the PLS approach for log *k’*IAM.

The model was built using the total set of OSCs, resulting in two statistically significant components (CPLS), whose parameters are detailed in Table 2. As shown in Figure 2, the agreement between the measured and calculated data from the derived PLS model is very satisfactory. There was also a linear tendency of the residuals when observing the normal probability plot of the said values (Data not shown), which indicates that the error terms were normally distributed.

**TABLE 2:** Statistical parameters of the PLS model resulting from OSC interactions with the IAM phase.

Comp	R2X (CUM)	R2Y(CUM)	Q^2^(CUM)	RMSS	F statistical
CPLS-1	0.452	0.749	0.712	8.60	175.46
CPLS-2	0.675	0.933	0.614		
(*R* ^2^ = 0.9335) (Q = 0.8886)					

The only compound that showed an outlier behavior was the AMS, which, while observing the standardized residuals, showed more than two standard deviations with respect to the centralized tendency of the rest of the compounds. Thus, considering this outlier, a second PLS model was built without AMS (compound 14), and as expected the PLS model's—with two components—statistical quality improved (*R* ^2^ = 0.953; Q = 0.763; and F = 240.76).

### QSAR Models to Assess Permeability Parameters for the OSCs Under Study

#### IAM Phase Ability to Assess OSC Human Intestinal Absorption

To evaluate the effectiveness of the IAM phase in the prediction of human intestinal absorption percentage (HIA%), we built several QSAR models based on MLR analysis. Since there is a lack of information regarding organosulfur compounds' HIA *in-vivo* values, we used calculated values obtained from the PreADMET web application. First, we assessed the reliability of PreADMET data in relation to our OSCs. As shown by Kar et al. (2018), to analyze how much a certain model’s prediction for a query compound can be trusted, in our case for the target OSCs, it is necessary to evaluate the applicability domain (AD) of that particular QSAR model. In this sense, it is important to recognize that QSARs are associated with restrictions in terms of the grouping of chemical structures, physicochemical properties, and mechanisms of action for which the models can produce reliable predictions. To evaluate the AD space of a specific model, AD approaches follow a range in descriptor values or distance-based hypothesis. Based on the previous concepts and considering the data set from Zhao et al., 2002, 241 molecules used to build the HIA-QSAR model that supports the PreADMET web server prediction values, we calculated several descriptors such as nHBAcc; nHBDon; MLogP; McGowan_Volume; TopoPSA; MW; AMW; and XLogP. We also calculated these parameters corresponding to our OSCs set. We then carried out a PCA, since principal components analysis has showed to be a valid approach to assess the AD space of a QSAR model (Kar et al., 2018). Results are shown in the Supplementary Material section. Shortly, it was possible to confirm OSCs belonging to the original data predictor space that supports PreADMET web server values. The Mahalanobis distance plot (Supplementary Figure S2) serves to probe the previous approach, where it can be seen that OSCs showed acceptable Mahalanobis distance. The Mahalanobis distance measures the distance from each point in multivariate space to the overall mean or centroid, utilizing the covariance structure of the data. All these results serve to assure that HIA predicted values of the OSCs under study are actually reliable to build the following QSAR models (Minitab v.17; Minitab Inc.; State College, PA 2010).

**FIGURE 3:**
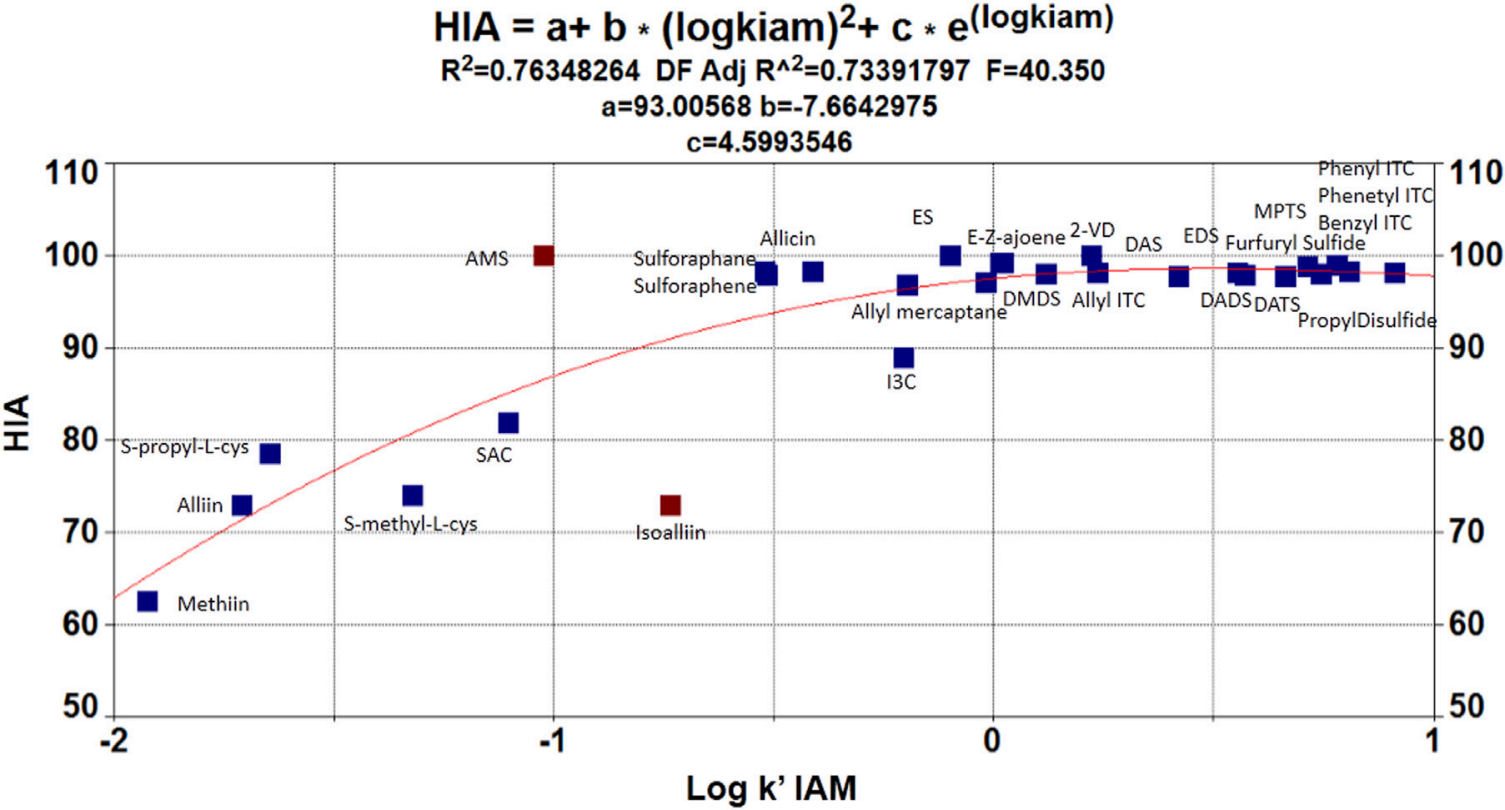
Nonlinear QSAR model showing the relationship between log *k’* (IAM) and (HIA %).

Focusing on our QSAR models, considering a linear estimation, a poor statistical correlation was found for the target OSCs (S = 6.04354; *R* ^2^ (adj) = 46.8%; PRESS = 371.285; F = 6.27). Hence, based on the previous results and OSC disposition when plotting log *k’* (IAM) vs. HIA (Figure 3), a nonlinear estimation was performed to model the data. In this case, employing TABLE 2D^®^ an exponential function was obtained with good statistical fitting. The resulting regression equation was:

$$\begin{array}{l} \mathrm{HIA}\left(\%\right)=\mathrm{93}\mathrm{.0}\left(2\mathrm{.494}\right)-7\mathrm{.66}\left(1\mathrm{.202}\right){\left[\log k’\left(\mathrm{IAM}\right)\right]}^{2}+4\mathrm{.60}\left(1\mathrm{.613}\right)e^{\left[\log k’\left(\mathrm{IAM}\right)\right]}\\ \left(0.000\right)\;\left(0.000\right)\;\left(0.009\right)\\ R^{2}=0.763;R^{2}\left(cv\right)=0.713;s=5.381;n=28;F=40.35 \end{array}$$ (3)

Even though a reasonable estimation was achieved to model OSC behavior regarding HIA, notably, a deviation in the fit was observed due to the behavior of ionizable OSCs (ACSOs, cysteine containing compounds, and I3C). In this sense, previous reports have treated ionizable compounds separately based on their lipophilicity values, and the occurrence of polar and/or electrostatic intermolecular interaction forces (Grumetto et al., 2015b; Russo et al., 2017). So, for better adjusting estimations, these compounds were separately modeled. In this case, a simple regression analysis and a fitted line plot were carried out for the seven ionizable compounds. As stated by Russo et al. (2017), the polar surface area is a critical factor to model HIA when dealing with ionizable compounds, since electrostatic and polar interaction forces affect the analyte–phospholipid partition. Therefore, the total polar surface area (named TPSA_1 to differentiate from the acronym TPSA—topological polar surface area—was considered in the model as follows.

$$\begin{array}{l} \mathrm{HI}A_{\mathrm{ionizable}\;\mathrm{OSCs}}\left(\%\right)=\mathrm{100}\left(4\mathrm{.182}\right)-0\mathrm{.143}\left(0\mathrm{.023}\right)\mathrm{TPSA\_1}\\ \left(0.000\right)\;\left(0.000\right)\\ R^{2}=0.884;R^{2}\left(cv\right)=0.837;s=3.087;n=7;F=38.20;W_{\mathrm{TPSA\_1}}=-0.940 \end{array}$$ (4)

**FIGURE 4:**
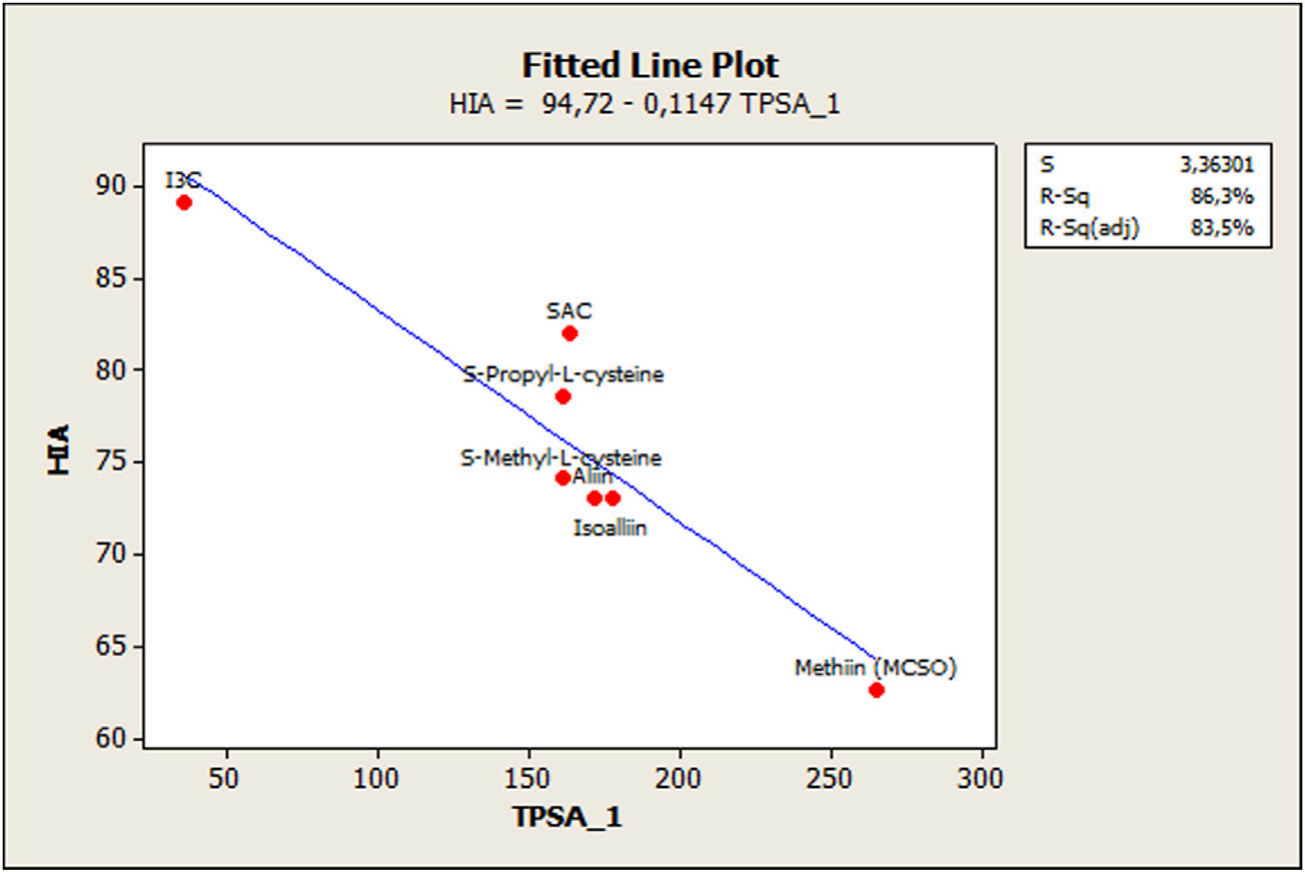
Scatterplot of ionizable OSCs showing the relationship in Figure 6. The relationship between actual and calculated log KP skin permeability values using the PLS approach.

Figure 4 shows the relationship between HIA % and total polar surface area (TPSA_1) for the following compounds: alliin, isoalliin, methiin, S-allyl-L-cysteine, S-methyl-L-cysteine, S-propyl-L-cysteine, and indole-3-carbinol.

#### Blood–Brain Barrier Permeation QSAR Modeling

Given that IAM chromatography has been successfully applied to drug permeability predictions across the blood–brain barrier (Luco and Marchevsky, 2006), a QSAR model based on IAM phase parameters was built to assess the BB barrier permeation of the target OSCs. Acknowledging that there is no BBB-empirical data reported for OSCs, the BBB-QSAR model was built using estimated data from the SwissADME web tool. The reliability of SwissADME BBB permeation data with regard to our OSCs was evaluated following the steps previously discussed in *IAM Phase Ability to Assess OSCs Human Intestinal Absorption*. Results from a PCA including several physico-chemical parameters (nHBAcc; nHBDon; MLogP; McGowan_Volume; TopoPSA; MW; AMW; and XLogP) are shown in the Supplementary Material section. The principal components analysis showed that our OSC data set actually belongs to the BBB-permeation predictor space generated from the 260 molecules used to developed BBB-permeation QSPR model by SwissADME (Daina and Zoete, 2016); therefore, OSC BBB-permeation data from SwissADME could be used to build the corresponding QSAR model.

BBB permeation data from the SwissADME web tool shows which compound could permeate the BBB (marked as Yes value, in Table 1) and which one could not (marked as No value, in Supplementary Table S1). Since BBB permeation was a categorical variable, a discriminant analysis using cross-validation was carried out. The grouping variable was BBB permeation, while the predictors were log *k’* (IAM) and TPSA. The discriminant ability was assessed by the correct classifications percentage, and the discriminant function quality was evaluated using the Wilks parameter, λ, which was obtained by a multivariate analysis of variance that tests the equality of group means for the variable in the discriminant model. The standardized discriminating function was

$$\begin{array}{l} DF_{\mathrm{standard}}=-0\mathrm{.716667 log}k\mathrm{’(IAM)+0}\mathrm{.901356}\;\mathrm{TPSA}\\ \mathrm{F=54}\mathrm{.425;Wilks}’\mathrm{lambda}\left(\lambda \right)=0.18678;P\left(value\right)=0.0000;n=28 \end{array}$$ (5)

**FIGURE 5:**
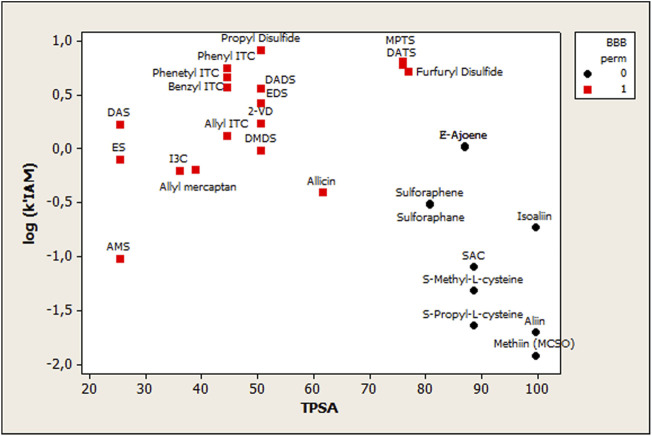
Scatterplot showing the relationship between log *k’* (IAM) vs. TPSA, considering OSC distribution using BBB permeation as the grouping classification variable. BBB permeating organosulfur compounds (group 1), and BBB not-permeating organosulfur compounds (group 0).

The DF equation is highly significant as *p* < 0.00001, and amongst the 28 observations used to fit the model, 28 (or 100.0%) were correctly classified. The correct classification rate was evaluated using cross-validation, and 100% was obtained (28 of 28). Figure 5 plots the relationship between log *k’* (IAM) vs. TPSA, considering OSC distribution using BBB permeation as the grouping classification variable. Therefore, it is possible to observe two different groups: BBB permeating organosulfur compounds (group 1) and BBB not-permeating organosulfur compounds (group 0).

#### IAM Phase Ability to Assess OSC Skin Permeability

To study the influence of OSC chemical structures on skin permeability a PLS analysis was carried out. For this purpose, several calculated values obtained from the SwissADME web tool were used, including log *K*_P_, a skin permeability coefficient resulting from a quantitative structure–property relationship model built by Potts and Guy (1992). As in the previous sections, we checked whether our OSC data set belonged to the data predictor space used to build *K*_*P*_ predictions values in the SwissADME web tool. These results are shown in the Supplementary Material section.

As an indicator of polarity and size, and given that these properties showed influence in other skin permeability *in silico* models (Guth et al., 2014), the TPSA descriptor was also considered in this model as a predictor. The other descriptors used here were Tm_1 (WHIM descriptor) and TE1 (Charge descriptor), which resulted from a stepwise regression analysis (SRA) and VIP (variable importance in the projection) parameter. Statistical significance of the QSRR-PLS derivatives models was evaluated by means of variance of the matrices X and Y (R2X and R2Y), the square correlation coefficient (*R* ^2^), standard deviation (RMSS), and the statistical F. The predictive ability was evaluated by the cross-validation coefficient (Q). The complete set of OSCs was used, resulting in two statistically significant components (CPLS), whose parameters are detailed in Table 3 **.** The obtained regression coefficients are shown in Table 4.

**TABLE 3:** Statistical parameters of the PLS model resulting from OSC log *K*_P skin permeability_ with the IAM phase and physico-chemical parameters.

Comp	R2X (CUM)	R2Y(CUM)	Q^2^(CUM)	RMSS	F statistical
CPLS-1	0.611	0.814	0.791	38.87	142.94
CPLS-2	0.902	0.920	0.897		
(*R* ^2^ = 0.9196) (Q = 0.897)					

**TABLE 4:** PLS-1 skin permeability regression coefficients.

	Log *K*_P_ (cm/s)	Log *K*_P_ (cm/s) standardized
Constant	−49.401	0.000
Log *k’* (IAM)	1.046	0.531
TPSA	−0.023	−0.349
TE1	−0.152	−0.293
Tm_1	0.112	0.237

**FIGURE 6:**
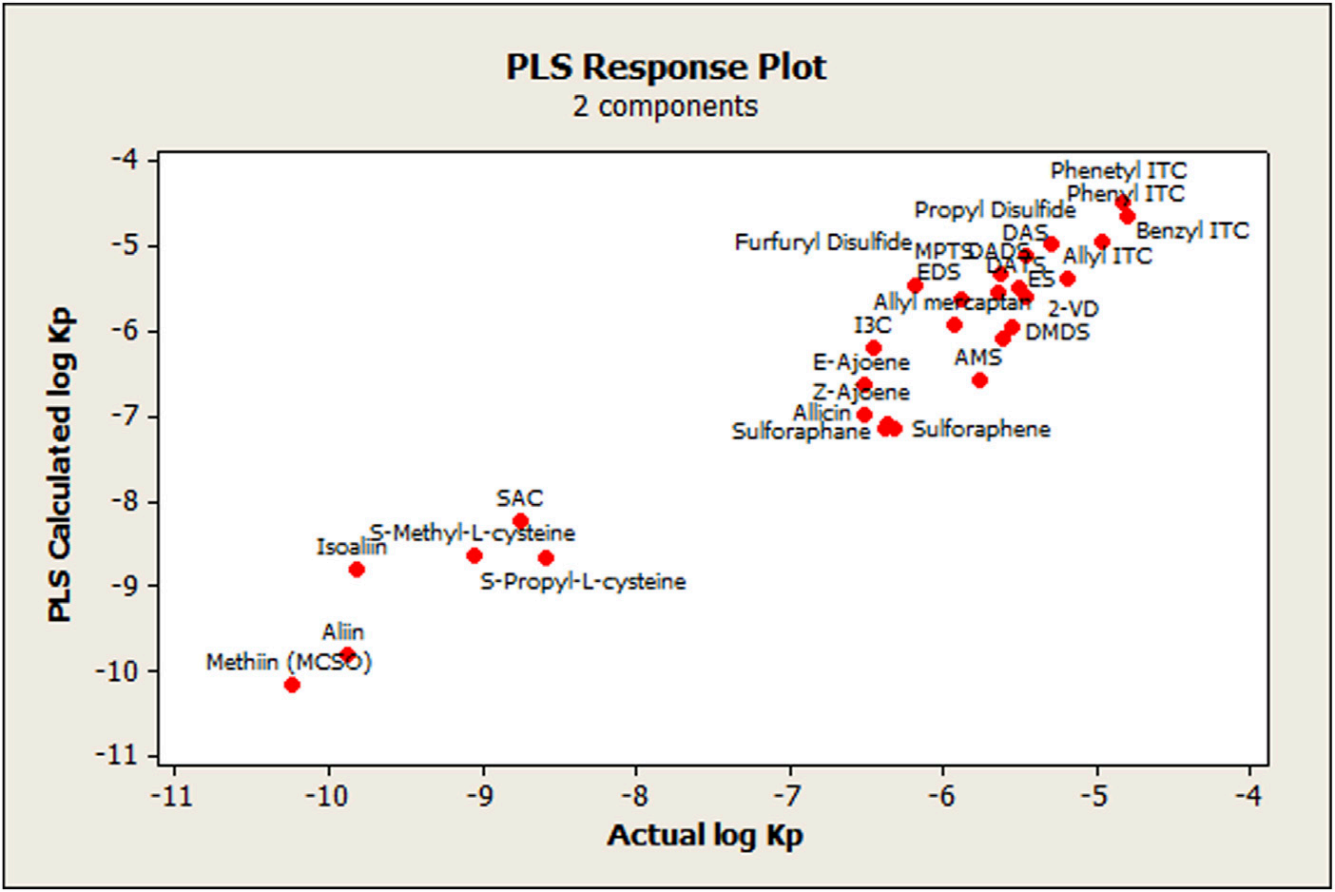
Relationship between actual and calculated log *K*_P__skin permeability_ values using the PLS approach.

Graphically, a high agreement between observed log *K*_P skin permeability_ values and the corresponding calculated ones derived from the PLS-1 model can be seen in Figure 6. There was also an approximately linear tendency of the residuals, when observing the normal probability plot of the said values (data not shown), which indicates that the error terms were normally distributed, except for isoalliin, which showed more than 2 standard deviations from the central tendency of the remaining OSCs.

## Discussions

The main purposes of the present study were, first, to build different QSRR models to obtain some insight into the mechanism of IAM retention for the 28 phytochemicals with sulfur-containing groups under study. Secondly, the present study aimed to establish whether, and to what extent, the IAM-chromatographic measures for the target OSCs, serve to assess ADME parameters. Given the chemical nature of the phospholipids that make up the IAM columns’ stationary phase, it was only logical to, first, study lipophilicity parameters associated with OSCs. It is well-known that passive absorption of orally consumed substances is related to drug lipophilicity, commonly expressed as the logarithm of the n-octanol/water partition coefficient, *log P*. And this partition phenomenon is a driving force for the membrane passage (Grumetto et al., 2015b). So, in order to evaluate IAM phase ability in assessing OSC lipophilicity, we studied the relation between **log *k’*IAM** values and calculated partition coefficient ***log P*** values**,** obtained from the SwissADME web tool (iLOGP). The regression model’s obtained results showed that even though there was a suitable fitting between the iLOGP and log k*’*IAM variables (both physicochemical parameters carry similar information regarding lipophilicity), they do not encode the same information regarding specific interactions, as shown below. This fact agrees with the other reports (Grumetto et al., 2015a; Russo et al., 2017) which proposed that when a compound is ionizable and/or presents a polar surface area ≠ 0, the interactions with the two partition phases, i.e., n-octanol and phospholipids, are differently affected by electrostatic and polar interaction forces. Thus, it may be concluded that the balance between polar and hydrophobic effects is not the same toward the n-octanol and IAM phases. Figure 1 supports this conclusion, where these “extra” intermolecular interactions become indirectly visible because of the observed divergence between the different classes of compounds. In view of this, it seems reasonable to consider a common interaction mechanism between the compounds containing sulfoxide groups, in which some of them, in turn, present charged groups and different p*K*a values (see Table 2) and the phospholipid constituents of the IAM stationary phase. This fact explains the modest fit observed between the log *k’* (IAM) and iLOGP parameters, indicating the octanol partition system’s inadequacy to mimic the complex interactions between the IAM phase and the ionized forms of the OSCs under study. At this point, it is important to highlight that we did not address log D evaluation because, at the experimental pH used during OSC chromatographic analysis, the log D and log P values were the same.

All these results gave us the guidelines to further explore other molecular descriptors to explain OSC-retention behavior in the IAM phase adequately. From a stepwise regression analysis and using the VIP (variable importance in the projection) parameter, different descriptors were selected, and an MLR, as well as a PLS model, were built based on the best subset of structural parameters. From the analysis of the results obtained, and as previously discussed, the specific polar interactions were better modeled by two electronic descriptors, the Mulliken electronegativity and the relative negative charge (RNCG_1). These results are in agreement with the findings by other authors, who have shown that the polar/electrostatic molecular properties affect the phospholipid–analyte partition (Grumetto et al., 2015b; Russo et al., 2017). The presence of rotatable bonds descriptor in the model requires a separate discussion. In the most general sense, it may be considered as conveying information about molecular flexibility, and interestingly, this property appears to be also involved in the IAM retention process. The molecular flexibility plays an important role when analyzing orally administered drugs, since increased rotatable bonds count (meaning a greater molecular flexibility) has been reported to negatively affect the permeation rate (Li et al., 2003). In this way, our results agree with the latter, since the negative sign of the rotatable bonds variable in the developed models suggests that the higher the molecular flexibility, the weaker the interaction with the IAM phase, and consequently, a shorter retention time could be observed. However, it must be considered that molecular rigidity is a much more complex issue than the simple counting of rotatable bonds. Thus, this molecular property’s appearance in the models suggests the importance of conformational constraints in the OSC-IAM partition.

Once the molecular factors that govern OSCs’ retention in the IAM column were elucidated, we assessed the predictive performance of IAM retention data on some permeability parameters obtained from the SwissADME web tool, and the PreADMET web-based application. Accordingly, the human gastrointestinal absorption percentage (HIA%), blood–brain barrier (BBB) permeation, and skin permeability (Log *K*_P_) were investigated. It should be highlighted that there are no experimental permeability data for these OSCs in the literature. To assure the reliability of these predicted values, we first checked our OSC data set belonging to each predictor space that supports the corresponding HIA, BBB passage, and *K*_*P*__*skin*_ values obtained from the web servers.

Regarding HIA (%), it has been previously shown by other authors that the nonlinear approaches outperform linear models in explaining HIA% data variance, since linear models tend to overestimate the HIA % for low permeable drugs, but this bias is not so marked in nonlinear models (Talevi et al., 2011). Given that an MLR model based on HIA (%) vs. log *k*' (IAM) showed a poor statistical correlation between the target compounds, and based on OSC disposition when plotting log *k’* (IAM) vs. HIA (%) (Figure 3), a nonlinear estimation was performed to model the data. As proposed by Talevi et al., the nonlinear estimation explained better the OSC-IAM partition. Moreover, Yen et al., 2005 have discussed that when molecules contain one or more ionizable groups, their lipophilicity changes with respect to pH resulting in a nonlinear relationship with gastrointestinal absorption. However, considering the results from the QSRR models and based on the OSC scatterplot of HIA (%) vs log *k’* IAM, it is possible to see that log *k’* (IAM) describes better the neutral compounds' behavior (Ermondi et al., 2018). But it is not possible to achieve a successful prediction of HIA permeability using only log *k’* IAM for all 28 OSCs.

A separate modeling of ionizable compounds shows that the total polar surface area (TPSA_1) explains better the behavior of sulfoxides- and cysteine-containing compounds, whose HIA permeation is reduced compared to the other OSCs. This descriptor has been already proven as a fundamental molecular parameter to describe the electrostatic/polar interaction in the absorption process (Grumetto et al., 2015b; Russo et al., 2017). Singh and Singh (2010) have found similar results with regard to HIA estimation of ACSOs, which showed moderate permeation capabilities, and they also discussed the importance of polar surface interactions in this matter. In short, membrane partitioning, during passive passage, has been exposed as a two-step process. In the first step, lipophilicity impulses the compounds into the phospholipid bilayer. On the other side, in the second step, the drug is transferred into the phospholipid bilayer’s interior, and the main interactions between the bilayer and polar parts of a solute are related to hydrogen bonding and polarity. Therefore, the permeation rate depends mainly on simple molecular descriptors such as hydrogen bond capacity, lipophilicity, and size and charge of the molecule. In this sense, the polar surface area (PSA), which is generally assumed to be related to hydrogen bonding capacity, becomes an excellent predictor of passive membrane permeability, especially for ionizable and/or polar compounds (Artursson and Bergstrom, 2003).

On another note, organosulfur blood–brain barrier permeation (OSC-BBB) was studied using a QSAR model based on the obtained IAM retention parameters. In this case, a linear discriminant analysis (LDA) was used to fit the classification of OSCs as BBB(+) or BBB(-) penetrators of the BBB. The use of the log k*’* (IAM) parameter was necessary to obtain a reasonable classificatory model, but it is not enough to fully explain the OSC-BBB permeation. Thus, the combination of log k*’* (IAM) and TPSA yielded the best OSC-BBB model classificatory. TPSA has also been related to BBB permeation models in several investigations (Luco, 1999; Luco and Marchevsky, 2006; Janicka et al., 2020) showing that the polar surface area is one of the most important factors determining the passage of molecules through the BBB.

Finally, the IAM phase ability to assess OSC skin permeability, expressed as the permeability coefficient Log *K*_P skin permeability_, was evaluated by a PLS model. As found in the previous models presented here, lipophilicity and polar interactions would appear to affect the skin permeation ability of the target OSCs, also in agreement with other authors (Barbato, 2006). But it is noteworthy that the other two molecular features seem to influence the skin biopartitioning phenomenon. Tm_1 (T total size index/weighted by mass) is a WHIM—weighted holistic invariant molecular—descriptor, that accounts for 3D molecular size and atom distribution (Todeschini and Gramatica, 1997), and TE1 is a topographic electronic descriptor. These descriptors, therefore, show the influence of molecular shape, size, and charge in OSC permeation through the skin.

In relation to ADME data about these compounds, it has been argued that allicin, one of the major bioactive garlic-related compounds, can easily permeate cell membranes of phospholipids bilayers, where it carries out its activity intracellularly and interacts with the SH group. Even though this has high permeability, allicin is rapidly metabolized and transformed into other active compounds. Other OSCs such as SAC, diallyl disulfide, and diallyl trisulfide have also shown high cellular permeability, although their permeability passage varies from each compound and tissue (Rahman, 2007). On another note, vinyldithiins' pharmacokinetic properties were investigated. 1,3-Vinyldithiin seems to be less lipophilic and was rapidly eliminated from serum, the kidney, and fat tissue, whereas 1,2-vinyldithiin is more lipophilic and showed a tendency to accumulate in fat tissues (Egen-Schwind et al., 1992). Torres-Palazzolo et al. (2018) showed that allicin, DAS, and 2VD would be able to permeate less than 5% through the intestine. Something that has been extensively discussed is the rapid reaction of OSCs with blood components, resulting in different metabolites with possible bioactive effects in the organism (Lawson and Gardner, 2005). To summarize, something to highlight from the models built here in relation to the different permeation capacities of the OSCs under study is the possibility of delving into the molecular mechanisms that govern these properties and ends up affecting the variations in OSC permeation rates in different tissues. We could observe the influence of the hydrophobic factors that govern OSC membrane passage, as well as the high effect of simple molecular descriptors such as hydrogen bond capacity, size, and charge of the molecules, particularly considering the polar surface area–related parameters, which greatly affect the ionizable compounds’ passage. It is necessary though, to delve deeper in organosulfur compounds’ ADME properties, for example, by comparing our results with cell-based and tissue-based models to validate the QSAR models proposed here for future uses.

## Conclusion

To assure OSC bioefficacy, a first evaluation must be conducted in terms of the pharmacokinetic potential of the said phytochemical agents. So far, our results proved that an *in silico*–based QSAR modeling was a convenient approach to assess different OSC permeability parameters. All this information serves to give an insight into the mechanisms implied during the OSC-membrane interaction. In brief, the evidence presented here suggests that in general lines, organosulfur compounds with sulfide groups showed a higher chromatographic retention using the IAM column, and accordingly, a higher permeation capability, which was mainly associated with lipophilic/polar interactions. On the contrary, ionizable OSCs showed a lower permeation capability as a result of different membrane–analyte interactions. Therefore, it may be concluded that the ADME properties under study are strongly dependent on hydrophobic factors as expressed by log *k’* (IAM), which provides evidence for the great potential of the IAM phases in the development of QSAR models.

## Data Availability

The original contributions presented in the study are included in the article/Supplementary Material; further inquiries can be directed to the corresponding authors.
