# Supplementary figures and images for: Permeability Data of Organosulfur Garlic Compounds Estimated by Immobilized Artificial Membrane Chromatography: Correlation Across Several Biological Barriers
Source: Front Chem. 2021 Sep 20;9:690707. doi: 10.3389/fchem.2021.690707 (PMC8488277; doi:10.3389/fchem.2021.690707)

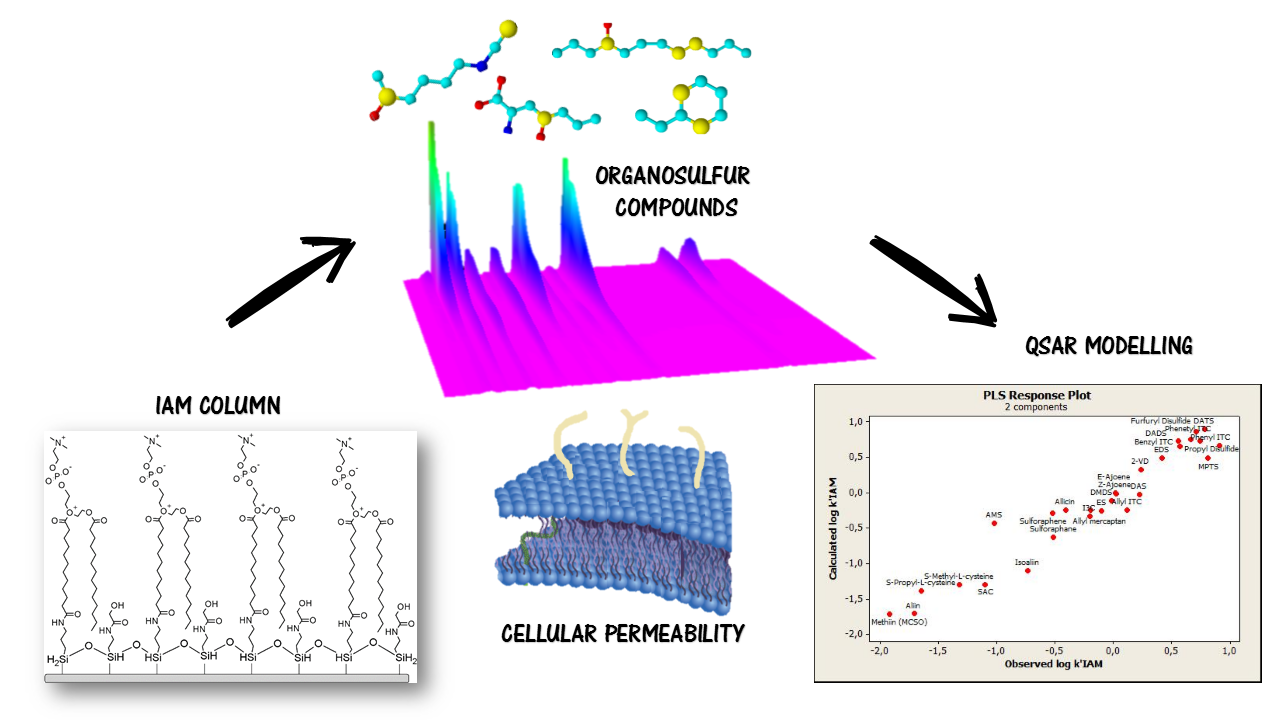

Supplement: Supplementary file 1 [file Image1.TIF]
