# Supplementary material for: Permeability Data of Organosulfur Garlic Compounds Estimated by Immobilized Artificial Membrane Chromatography: Correlation Across Several Biological Barriers
Source: Front Chem. 2021 Sep 20;9:690707. doi: 10.3389/fchem.2021.690707 (PMC8488277; doi:10.3389/fchem.2021.690707)
Supplement: Supplementary file 2 [file DataSheet1.PDF]

Supplementary Material

Table S1. Organosulfur compounds understudy and their chemical structure.

N°	Name	Chemical structure
1	Propyl disulfide	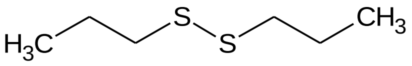
2	Isoalliin	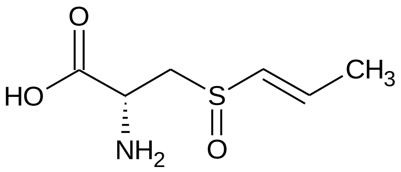
3	Alliin	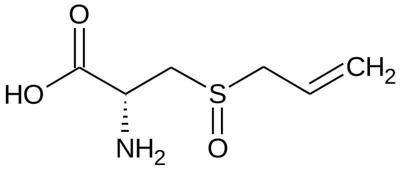
4	S-allyl-L-cysteine	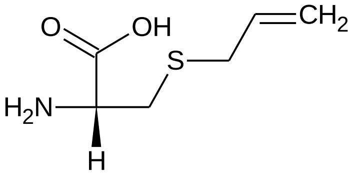
5	S-propyl-L-cysteine	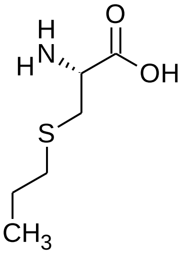
6	S-methyl-L-cysteine	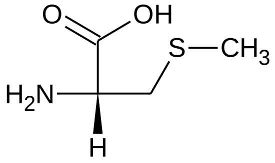
7	Methiin	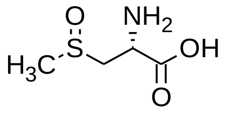
8	2-Vinyl-4H-1,3-dithiine	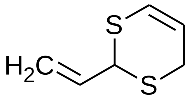
9	Diallyl disulfide	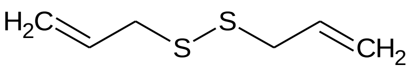
10	Ethyl disulfide	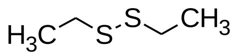

11	Dimethyl disulfide	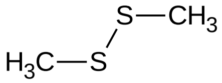
12	Ethyl sulfide	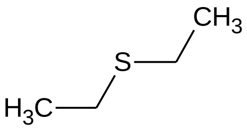
13	Allyl methyl sulfide	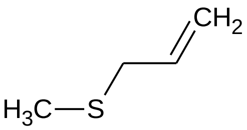
14	E-Ajoene	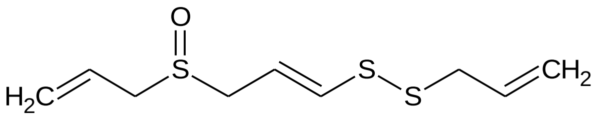
15	Z-Ajoene	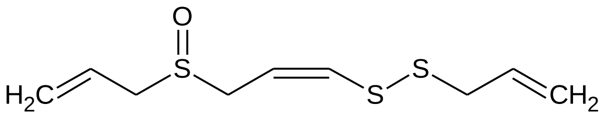
16	Diallyl sulfide	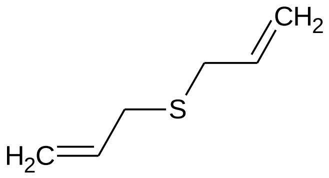
17	Methyl Propyl Trisulfide	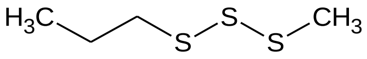
18	Furfuril Disulfide	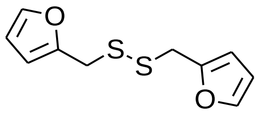
19	Allicin	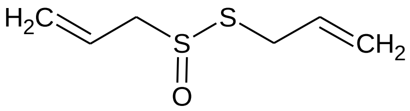
20	Allyl mercaptan	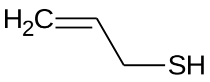
21	Sulforaphane	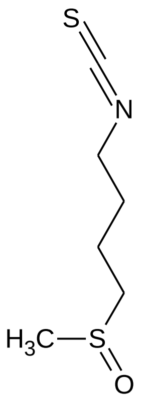
22	Sulforaphene	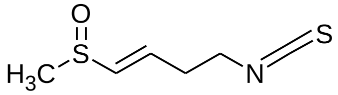

23	Diallyl Trisulfide	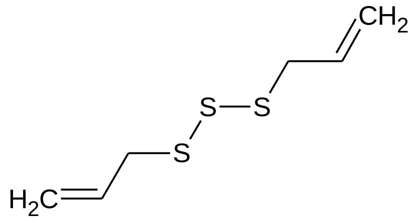
24	Indole-3-carbinol	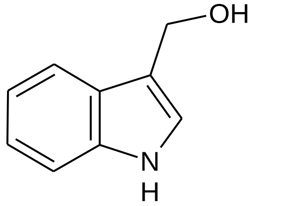
25	Allyl isothiocyanate	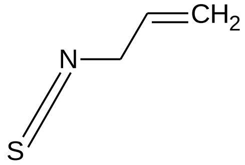
26	Phenetyl isothiocyanate	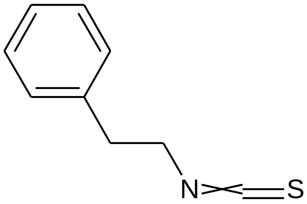
27	Phenyl isothiocyanate	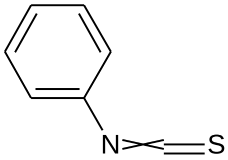
28	Benzyl isothiocyanate	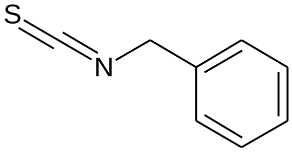

Results from the study of SwissADME and preADMET data reliability

- **HIA Evaluation**

We assessed the reliability of preADMET data in relation to our OSCs. Based on the data set from Zhao et al., 2002 **-241 molecules-** used to build the HIA-QSAR model that supports preADMET webserver prediction values, we calculated several descriptors such as nHBAcc; nHBDon; MLogP; McGowan_Volume; TopoPSA; MW; AMW; and XLogP. We also calculated these parameters corresponding to our OSCs set. We then carried out a PCA. Note: 3D Optimization for all compounds (dataset-HIA and OSCs) was carried out by using the OpenBabelGUI with the MMFF94 Force Field.

Results are shown as follows:

Table S2: Eigenanalysis of the Correlation Matrix (HIA predictor space from preADMET)

Eigenvalue	4.0985	2.4950	0.9432	0.1871	0.1722	0.0698	0.0285	0.0057
Proportion	0.512	0.312	0.118	0.023	0.022	0.009	0.004	0.001
Cumulative	0.512	0.824	0.942	0.965	0.987	0.996	0.999	1.000

Table S3: Eigenvectors of PCA (HIA predictor space from preADMET)

Variable	PC1	PC2	PC3	PC4	PC5	PC6	PC7	PC8
nHBAcc	0.470	-0.111	-0.040	0.008	-0.360	-0.752	-0.263	0.016
nHBDon	0.410	-0.222	0.266	-0.336	0.751	-0.133	0.127	0.016
MLogP	0.142	0.584	0.041	0.410	0.367	0.030	-0.576	-0.024
McGowan_Volume	0.419	0.322	-0.104	0.077	-0.115	0.156	0.383	0.721
TopoPSA	0.443	-0.231	-0.041	-0.315	-0.250	0.596	-0.480	-0.041
MW	0.441	0.232	-0.244	0.159	-0.054	0.113	0.438	-0.678
AMW	-0.024	-0.333	-0.862	0.166	0.304	-0.026	-0.099	0.127
XLogP	-0.157	0.525	-0.334	-0.748	0.009	-0.149	-0.063	-0.044

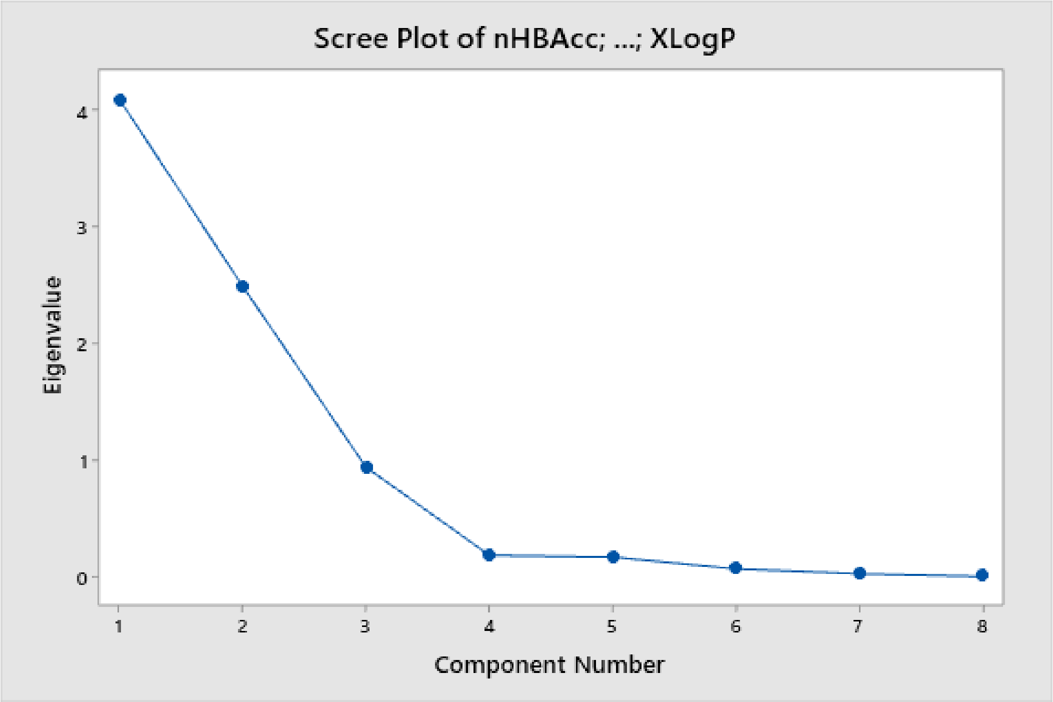

Figure S1: Scree plot – PCA nHBAcc; nHBDon; MLogP; McGowan_Volume; TopoPSA; MW; AMW; and XLogP (HIA predictor space from preADMET)

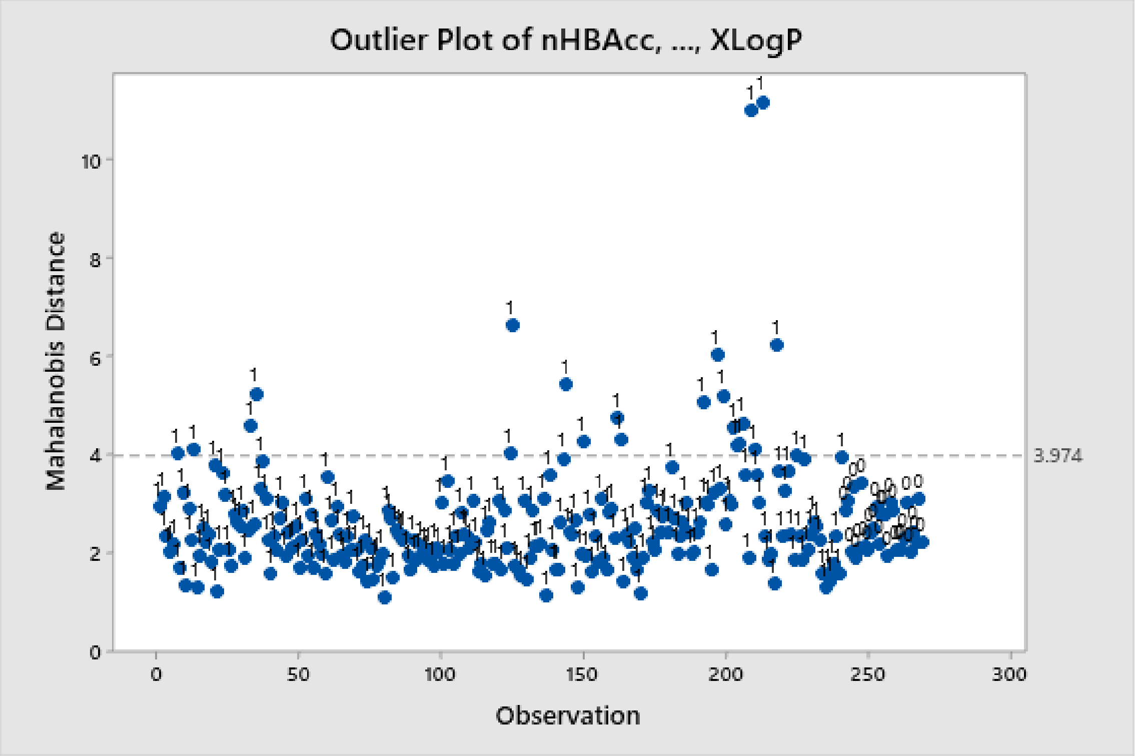

Figure S2: Outlier plot – PCA nHBAcc; nHBDon; MLogP; McGowan_Volume; TopoPSA; MW; AMW; and XLogP (HIA predictor space from preADMET).

Note: The Mahalanobis distance measures the distance from each point in multivariate space to the overall mean or centroid, utilizing the covariance structure of the data. Minitab displays a reference line on the outlier plot to identify outliers with large Mahalanobis distance values. The reference line is defined by the following formula:

When n – p – 1 is 0, Minitab displays the outlier plot without the reference line.

$$\sqrt{p\ x\ F\ inverse\ CDF\ (.95, p, n - p - 1)}$$

<u>Notation:</u> Y_i: data value vector at row; $\overline{Y}$: mean vector; S^{-1}: inverse of the covariance matrix; p: the number of variables; n: the number of non-missing rows.

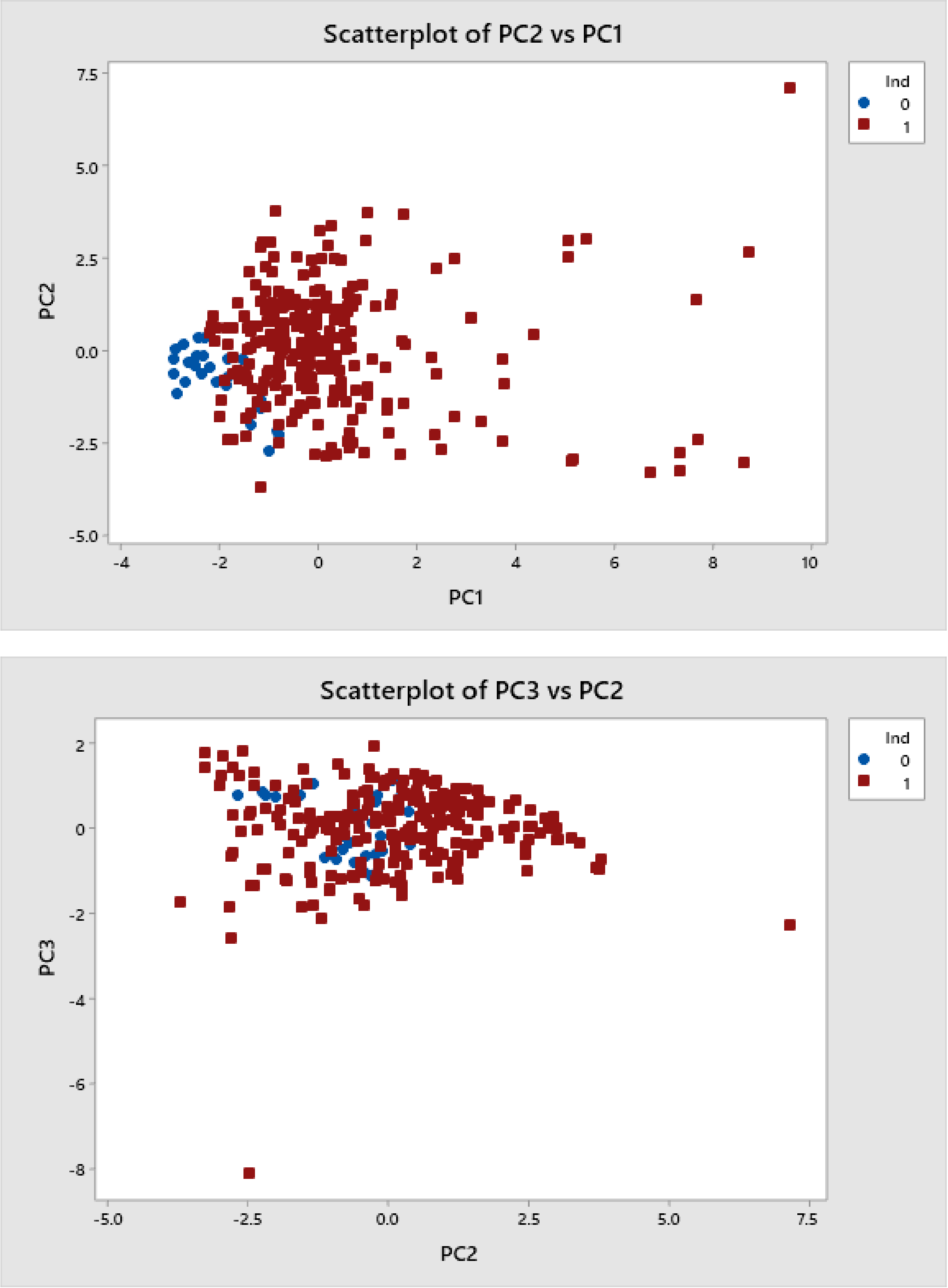

Figure S3: Scatter plots – PCA, showing PC1 and PC2, PC2 and PC3. Variables: nHBAcc; nHBDon; MLogP; McGowan_Volume; TopoPSA; MW; AMW; and XLogP (HIA predictor space from preADMET). CODE: 1= data set; 0= OSCs.

REFERENCES

The developed (%)HAI-absorption QSPR model by PRE-ADMET was implemented from "Evaluation of Human Intestinal Absorption Data and Subsequent Derivation of a Quantitative Structure Activity Relationship (QSAR) with the Abraham Descriptors", Zhao,Y.H. et al. J. Pharm. Sci. **2001**, 90, 749. The HIA-data set analyzed by PCA was obtained from this work (**241** compounds).

- **BBB permeation**

We assessed the reliability of SwissADME data in relation to our OSCs. Based on the data set from Daina and Zoete, 2016 **-260 molecules-** used to build the HIA-QSAR model that supports SwissADME webserver prediction values, we calculated several descriptors such as nHBAcc; nHBDon; MLogP; McGowan_Volume; TopoPSA; MW; AMW; and XLogP. We also calculated these parameters corresponding to our OSCs set. We then carried out a PCA. Note: 3D Optimization for all compounds (dataset-BBB and OSCs) was carried out by using the OpenBabelGUI with the MMFF94 Force Field.

Results are shown as follows:

Table S4: Eigenanalysis of the Correlation Matrix (BBB predictor space from SwissADME)

Eigenvalue	3.2527	2.6997	1.0352	0.4995	0.2864	0.1735	0.0455	0.0074
Proportion	0.407	0.337	0.129	0.062	0.036	0.022	0.006	0.001
Cumulative	0.407	0.744	0.873	0.936	0.972	0.993	0.999	1.000

Table S5: Eigenvectors of PCA (BBB predictor space from SwissADME)

Variable	PC1	PC2	PC3
nHBAcc	0.314	0.424	-0.002
nHBDon	0.093	0.447	-0.349
MLogP	0.455	-0.293	-0.121
McGowan_Volume	0.550	-0.031	0.016
TopoPSA	0.151	0.543	0.074
MW	0.540	0.041	0.145
AMW	-0.082	0.209	0.881
XLogP	0.251	-0.440	0.247

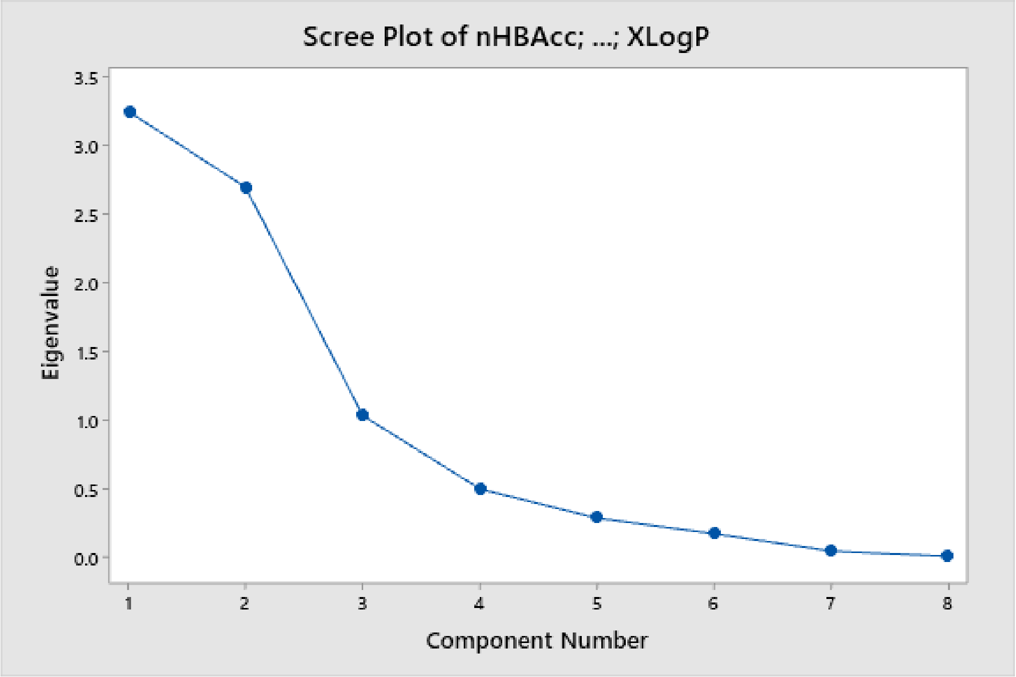

Figure S4: Scree plot – PCA nHBAcc; nHBDon; MLogP; McGowan_Volume; TopoPSA; MW; AMW; and XLogP (BBB predictor space from SwissADME)

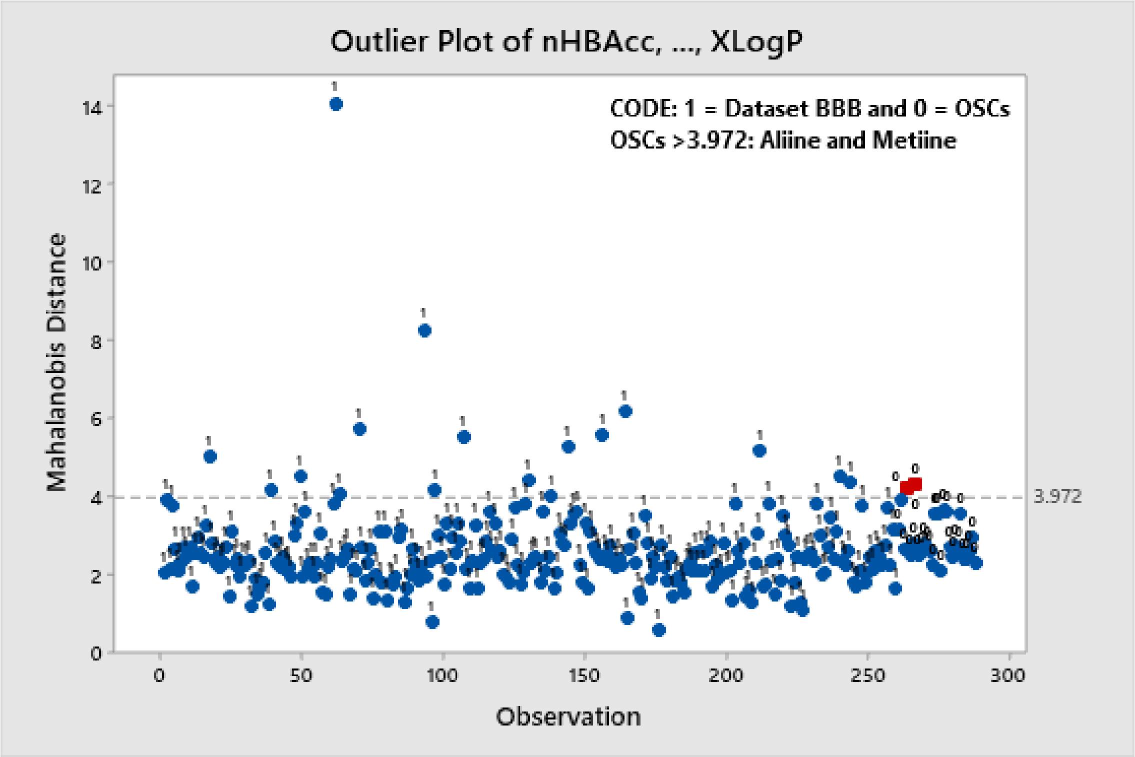

Figure S5: Outlier plot – PCA nHBAcc; nHBDon; MLogP; McGowan_Volume; TopoPSA; MW; AMW; and XLogP (BBB predictor space from SwissADME).

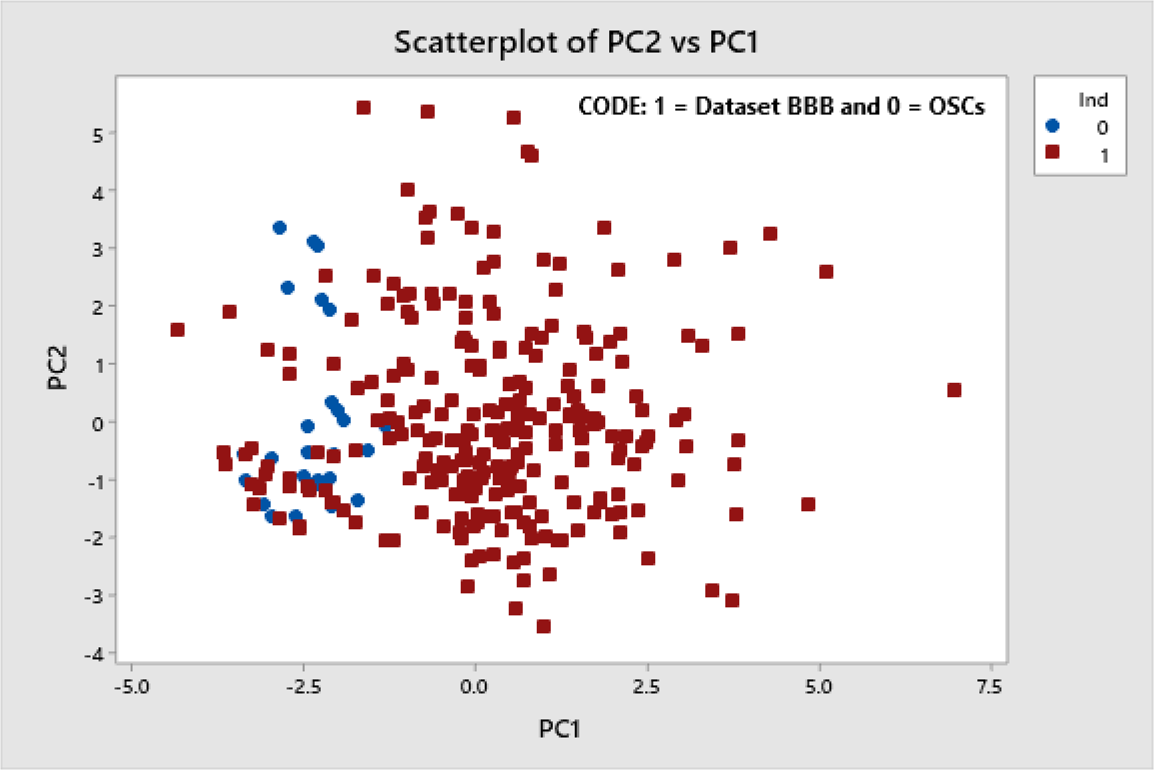

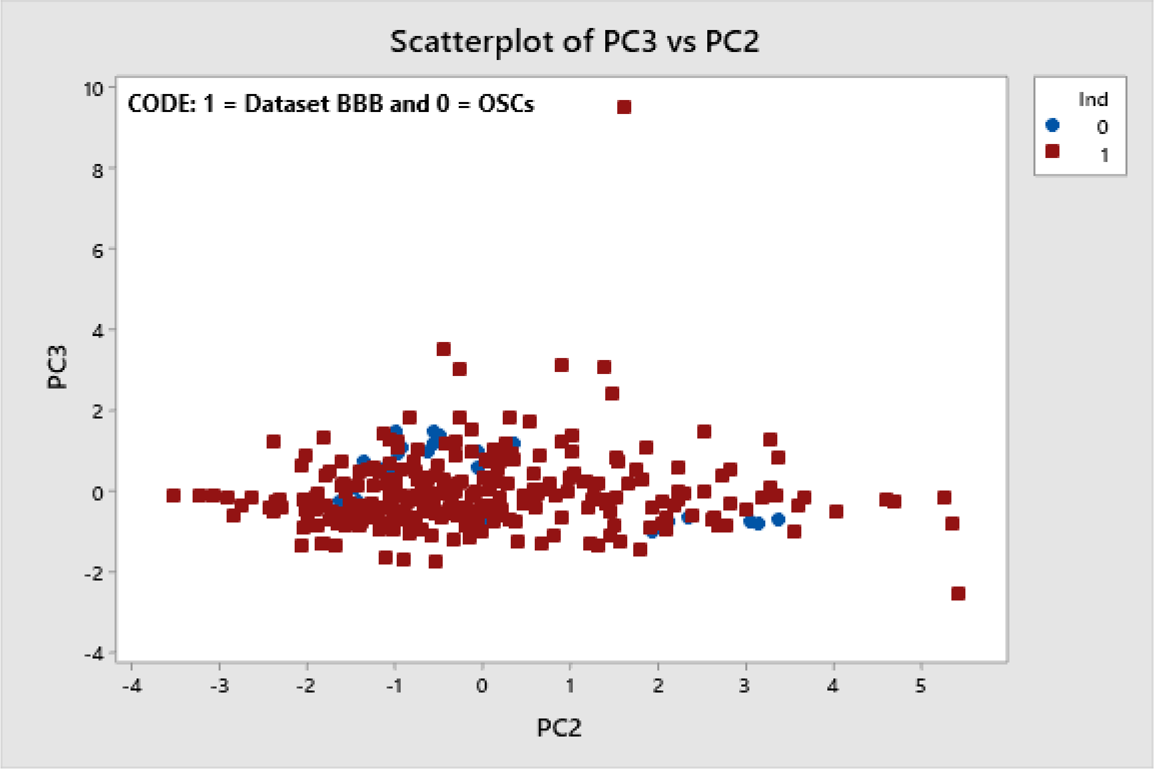

Figure S6: Scatter plots – PCA, showing PC1 and PC2, PC2 and PC3. Variables: nHBAcc; nHBDon; MLogP; McGowan_Volume; TopoPSA; MW; AMW; and XLogP (BBB predictor space from SwissADME). CODE: 1= data set; 0= OSCs.

REFERENCES

The developed BBB-permeation QSPR model by SwissADME was implemented from "A BOILED-Egg to predict gastrointestinal absorption and brain penetration of small molecules". Daina and Zete (**2016**) ChemMedChem 11(11):1117-1121. The BBB-dataset was obtained from this work.

- <u>**Skin permeation**</u>

We assessed the reliability of SwissADME data in relation to our OSCs. Based on the data set from Kladt et al., 2018 **-41 molecules-** used to build the HIA-QSAR model that supports SwissADME webserver prediction values, we calculated several descriptors such as nHBAcc; nHBDon; MLogP; McGowan_Volume; TopoPSA; MW; AMW; and XLogP. We also calculated these parameters corresponding to our OSCs set. We then carried out a PCA. Note: 3D Optimization for all compounds (dataset-BBB and OSCs) was carried out by using the OpenBabelGUI with the MMFF94 Force Field.

Results are shown as follows:

Table S6: Eigenanalysis of the Correlation Matrix (skin permeation predictor space from SwissADME)

Eigenvalue	4.4307	1.8567	1.0054	0.3225	0.2627	0.1053	0.0150	0.0017
Proportion	0.554	0.232	0.126	0.040	0.033	0.013	0.002	0.000
Cumulative	0.554	0.786	0.912	0.952	0.985	0.998	1.000	1.000

Table S6: Eigenvectors of PCA (skin permeation predictor space from SwissADME)

Variable	PC1	PC2	PC3	PC4	PC5	PC6	PC7	PC8
nHBAcc	0.433	0.185	0.140	0.104	-0.171	-0.833	-0.155	0.021
nHBDon	0.348	0.316	0.227	-0.802	0.248	0.123	0.078	-0.034
MLogP	0.370	-0.420	-0.011	0.087	0.460	0.139	-0.665	0.080
McGowan_Volume	0.451	-0.190	-0.126	0.186	0.062	0.094	0.370	-0.749
TopoPSA	0.368	0.361	-0.184	0.093	-0.617	0.442	-0.339	0.022
MW	0.455	-0.118	-0.202	0.161	0.095	0.098	0.517	0.653
AMW	-0.093	0.299	-0.874	-0.119	0.279	-0.186	-0.094	-0.061
XLogP	0.001	-0.649	-0.273	-0.505	-0.476	-0.152	-0.010	0.022

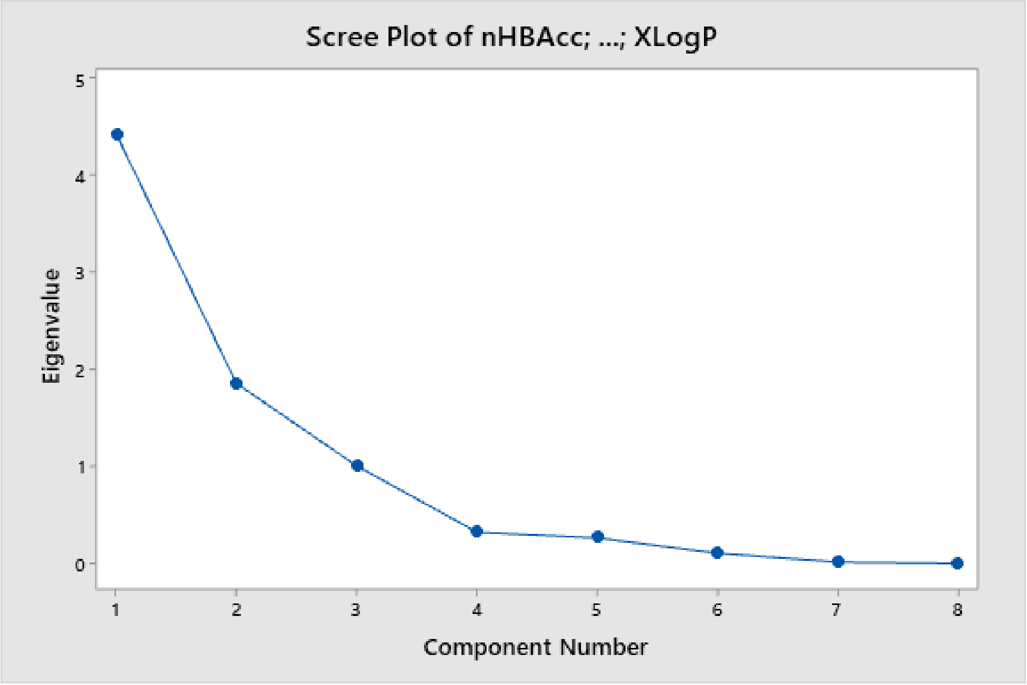

Figure S7: Scree plot – PCA nHBAcc; nHBDon; MLogP; McGowan_Volume; TopoPSA; MW; AMW; and XLogP (K_p predictor space from SwissADME)

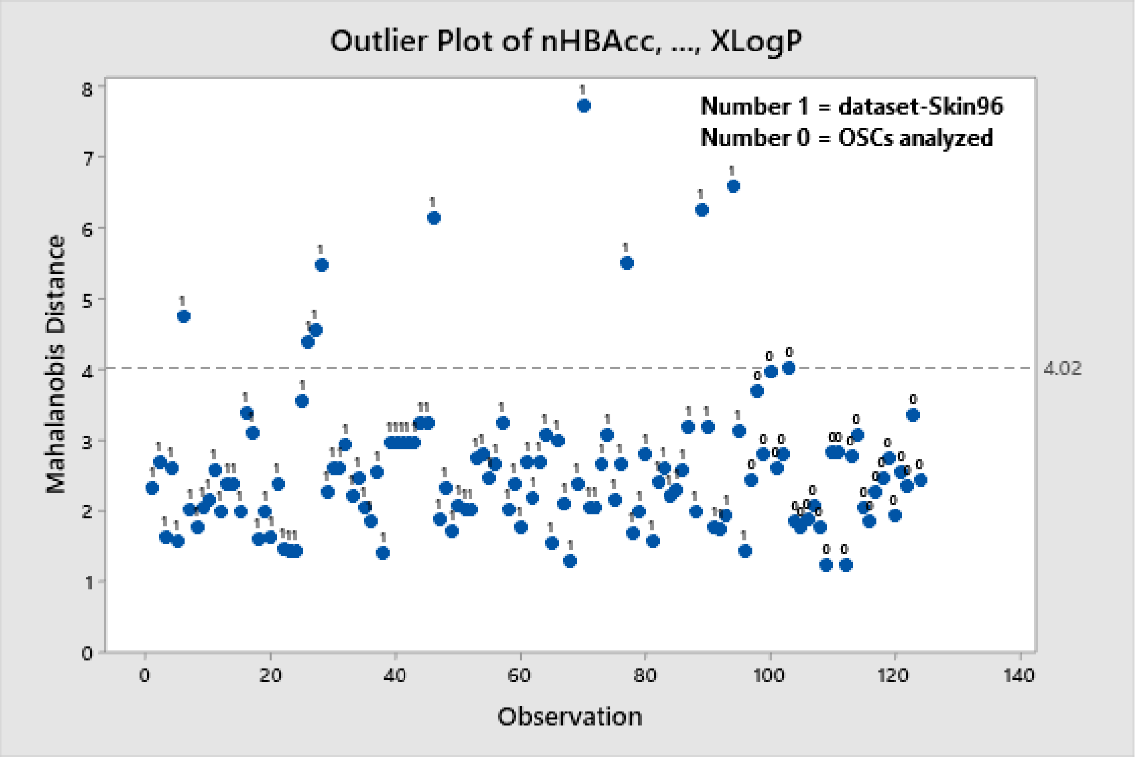

Figure S8: Outlier plot – PCA nHBAcc; nHBDon; MLogP; McGowan_Volume; TopoPSA; MW; AMW; and XLogP (K_p predictor space from SwissADME).

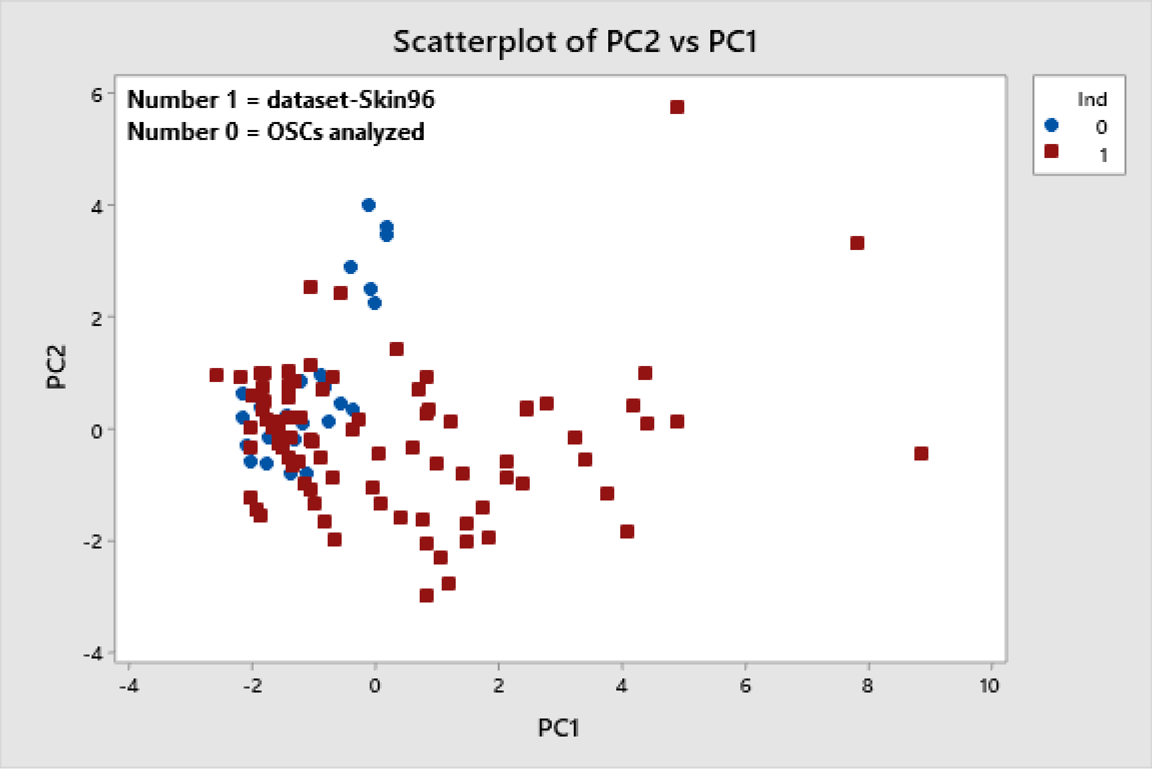

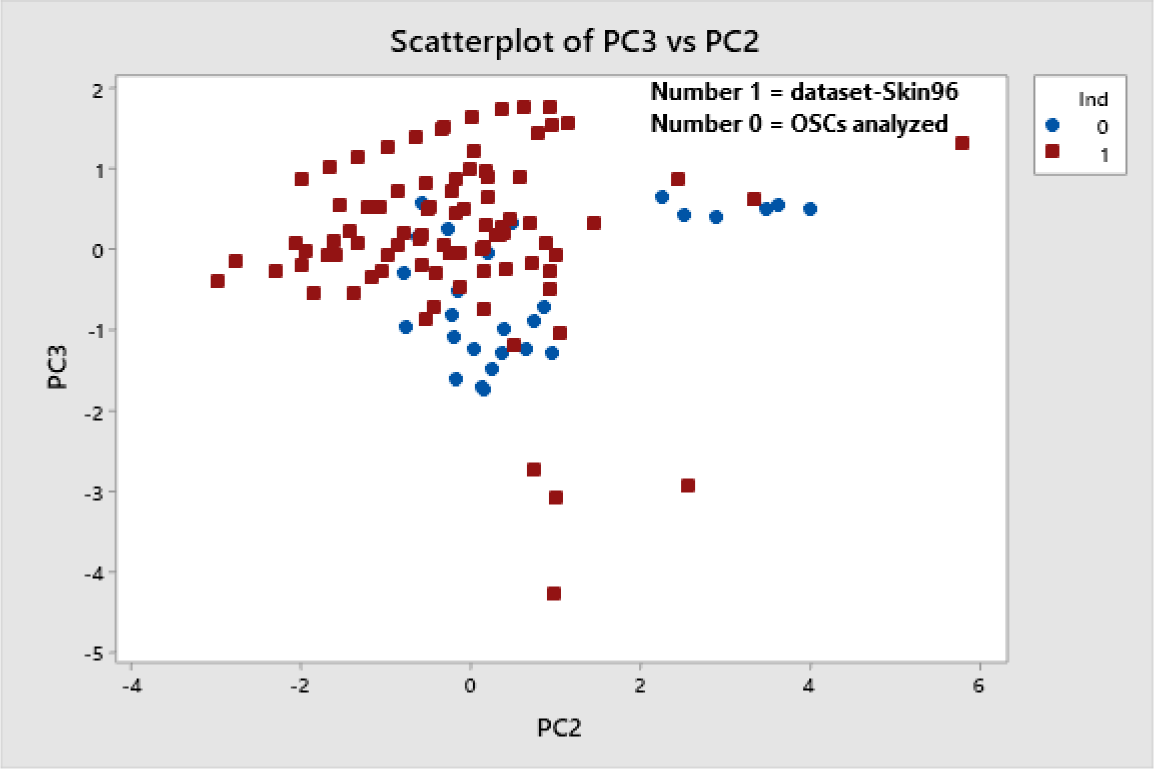

Figure S9: Scatter plots – PCA, showing PC1 and PC2, PC2 and PC3. Variables: nHBAcc; nHBDon; MLogP; McGowan_Volume; TopoPSA; MW; AMW; and XLogP (Kp predictor space from SwissADME). CODE: 1= data set; 0= OSCs.

REFERENCES

The developed skin-permeation QSPR model by SwissADME was implemented from Potts and Guy RH; Pharm. Res. The dataset used by Potts and Guy was the Flynn's dataset, which was obtained from: Kladt, C., Dennerlein, K., Göen, T. et al. Evaluation on the reliability of the permeability coefficient (Kp) to assess the percutaneous penetration property of chemicals on the basis of Flynn's dataset. Int Arch Occup Environ Health 91, 467–477 (2018). https://doi.org/10.1007/s00420-018-1296-5
